# Wildland-Urban Interface (WUI) Smoke Yields of Nonmethane Organic Gases from Combustion of Small-Scale Residential Building Surrogates

**DOI:** 10.1021/acsestair.5c00187

**Published:** 2025-10-09

**Authors:** Michael F. Link, Aika Y. Davis, Nathan M. Lima, Ryan L. Falkenstein-Smith, Rodney A. Bryant, Thomas G. Cleary, Dustin Poppendieck

**Affiliations:** National Institute of Standards and Technology, Gaithersburg, Maryland 20899, United States

**Keywords:** wildland-urban interface, structure fire, smoke yields, nonmethane organic gases

## Abstract

At the wildland-urban interface (WUI) structural fires can generate nonmethane organic gases (NMOGs) from burning urban fuels like structural lumber, plastics, and carpet. These NMOGs can contaminate nearby homes and affect indoor air quality. NMOGs have been quantified extensively from biomass burning, but few measurements exist of yields from structure fires. We calculated yields of 201 NMOGs generated from burning small-scale residential building surrogates. We also constructed surrogates of different sizes and stick packing densities to modulate air ventilation and simulate how reduced oxygen conditions in enclosed fires might affect NMOG yields. We find that reduced aromatics (e.g., benzene, naphthalene) show notably higher yields from combustion of the surrogates compared to biomass, whereas oxygenated NMOG (e.g., formaldehyde, acetaldehyde) yields are lower. Using factor analysis of NMOG time series, we observe chemical signatures from the combustion of synthetic polymers, wood, and mixed fuel char. Though we do not identify unique tracers we identify NMOGs that, if present in enhanced concentrations, may indicate WUI fire contamination in homes.

## Graphical abstract

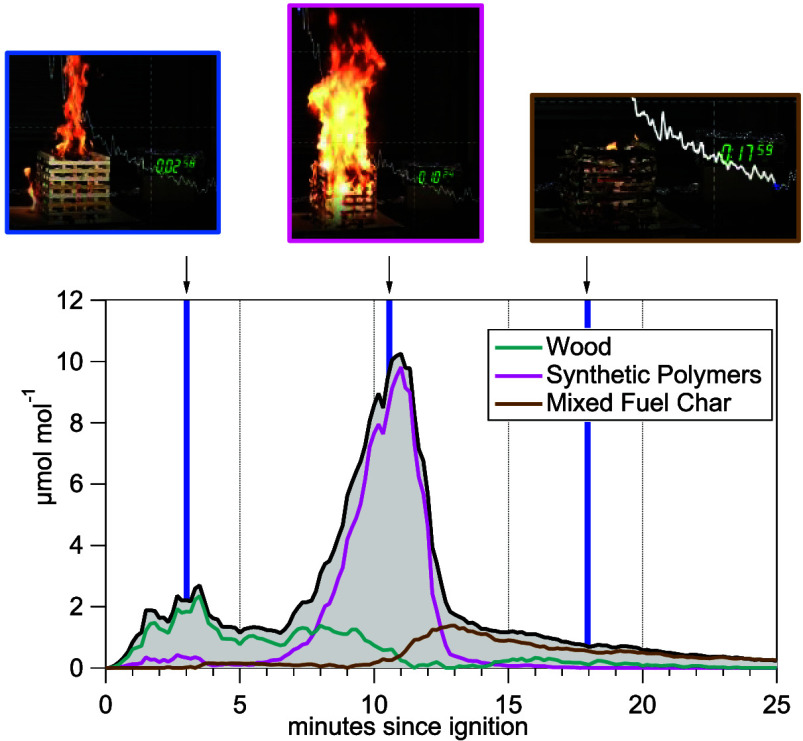

## Introduction

Over the past several decades in the United States wildfire frequency has increased coincident with an increasing encroachment of urban or suburban developments on natural areas thus increasing the risk of so-called wildland-urban interface (WUI) fires.^1,2^ The fuel of WUI fires is distinct from wildland fires and includes components like structures, vehicles, and furnishings—in addition to biomass.^4^ Residential buildings are an important fuel source for WUI fires, the smoke of which can contaminate other buildings near and regionally downwind of the fire.^5–7^ The primary components of residential buildings include treated and untreated lumber, plastics (used in siding, plumbing, electrical components), insulation (e.g., fiberglass, spray foam), composite materials (e.g., shingles, gypsum board), and the contents of the buildings themselves.^8^ Recently, Holder et al. demonstrated that the smoke from structural fires contains higher concentrations of heavy metals, dioxins, and polycyclic aromatic hydrocarbons (PAHs) compared to smoke from the burning of vegetation (i.e., biomass). Still, measurements of smoke from structural fires in laboratory or field settings are scarce. Quantified emissions from structure fires are needed to understand the impacts of WUI fires on air quality and health.^9,10^

Nonmethane organic gases (NMOGs) are key smoke constituents that impact outdoor and indoor air quality on local and regional scales.^11^ Research on NMOG emissions from biomass burning^12^ is extensive relative to WUI fuels—particularly for emissions from combustion of full-scale residential structures.^13^ Emissions from residential buildings consumed by WUI fires are difficult to estimate because the building itself is a mixed fuel with both structural (e.g., frame and siding) and internal components (e.g., furnishings, wirings, and paints) of variable chemical composition.

Emissions from structure fires can also be dependent on air ventilation because combustion can be contained and oxygen-limited with nonflaming conditions resulting in production of incomplete combustion intermediates.^14^ For example, enclosed areas in a structure (e.g., room, closet) adjacent to a well-ventilated fire can increase in temperature (through radiative and convective heat transfer) and create an excess of unburned fuel (pyrolyzate) in the air. Upon injection of oxygen-rich air, smoke and pyrolyzate can ignite in so-called “flashover events”.^15^ Similarly, oxygen availability may be limited for pyrolysis that occurs in the remains of a burned structure.^16^ If stoichiometric equivalence between fuel and oxygen is not achieved or is not in excess of oxygen, incomplete combustion will produce smoke products whose identity and yield are in part determined by the availability of oxygen.^17^

Small-scale experimental designs use apparatus like tube furnaces or cone calorimeters to quantify emissions by combusting individual components of residential buildings, such as lumber, plastics, and carpet.^18,19^ It is unclear to what extent these small-scale experiments represent likely emissions from a typical structure—particularly for smoke constituents other than CO and CO_2_.^20,21^ Some measurements have been done on full-scale structures, but these experiments are difficult because of resource demands.^22^

To address the need for more quantified emissions from WUI structures, we have designed and tested small-scale surrogates for residential buildings, suitable for flaming combustion experiments (i.e., combustion performed under a hood calorimeter), that can be used to produce smoke characteristic of a WUI fire.^8,23^ These surrogates can be configured with different fuels, thus providing experimental flexibility typically only available to small-scale experiments and opportunities to study fire dynamics (i.e., ignition method, ventilation effects) typically afforded to building-scale experiments. We present hood calorimeter measurements of 201 NMOGs emitted from the combustion of our mixed-fuel surrogates for an idealized residential building (hereafter referred to as “surrogates”). Additionally, we demonstrate how the surrogate composition can generate distinct NMOG signatures that may appear in elevated air concentrations in homes contaminated by real WUI fires.

## Methods and Materials

### Residential Building Surrogates

The construction method, combustion properties (e.g., burn time, heat release rate, smoke extinction, etc.), and select smoke emissions from the surrogates are described in Davis et al. Surrogates were constructed based on a design first described in Bryner and Mulholland who adjusted the packing density of sticks in the surrogates to simulate blast damage to a building from a nuclear explosion.^23^ Surrogate design features and composition are presented in Table 1.

**Table 1:** Residential Building Surrogate Composition and Properties

surrogate composition				
material	total mass percent (combustible mass percent)	structural use	chemical composition	example expected gaseous emissions
gypsum board (GYP)^a^	45.7 (n/a)	interior walls and ceilings	CaSO_4_ · 2H_2_O	SO_2_
untreated spruce-pine-fir (SPF)	21.6 (39.7)	structural frame	cellulose, hemicellulose, lignin	furans, carbonyls, reduced aromatics
oriented straight board (OSB)	14.8 (27.2)	wall, floor, roof	same as SPF treated with waterproof resin	furans, carbonyls, reduced aromatics
polyvinyl chloride (PVC)	7.0 (12.8)	pipes, electrical insulation, siding, and flooring	vinyl chloride polymer	vinyl chloride, hydrochloric acid (HCl)^b^, Cl radicals, Cl_2_
polyurethane rubber	5.9 (10.8)	surrogate for carpet/furniture upholstery, insulation	polymer from methylene diphenyl isocyanate and ethylene glycol	cyclic reduced aromatics, nitriles, isocyanates
acrylonitrile-butadiene-styrene (ABS)	5.1 (9.5)	plastic household goods	acrylonitrile, 1,3-butadiene, styrene polymer	monomer constituents

surrogate design and combustion					
surrogate ID	size	packing density	ventilation factor, ψ (cm)	total heat release (MJ)^c^	modified combustion efficiency MCE = ΔCO_2_/(ΔCO_2_ + ΔCO)^c^
SL	small	low	0.145	68 ± 1	0.927 ± 0.003
SM	small	medium	0.074	89 ± 3	0.947 ± 0.003
SH	small	high	0.014	114 ± 4	0.921 ± 0.002
LL	large	low	0.187	315 ± 3	0.952 ± 0.004
LM	large	medium	0.074	446 ± 4	0.925 ± 0.003
LH	large	high	0.020	500 ± 20	0.932 ± 0.006

a Gypsum is noncombustible.

b HCl was not measured in this study.

c Standard deviation (2σ) of three measurements. Δ indicates background-subtracted values.

Surrogates were constructed using sticks of six different component materials (GYP board, untreated SPF wood, OSB, PVC, polyurethane, and ABS) arranged into a rectangular prism. Sticks were arranged in perpendicular rows randomly but varied enough such that there were not multiple sticks side-by-side of the same material in rows. The initial mass of small surrogates was between 6 kg (small low density) and 13 kg (small high density), and large surrogates were between 33 kg (large low density) and 55 kg (large high density). Surrogate dimensions were 30.0 cm × 30.0 cm × 28.6 cm for small surrogates and 47.5 cm × 47.5 cm × 45.7 cm for large surrogates. Approximately 70% of the combustible mass fraction was wood (SPF and OSB) and 30% was synthetic polymers (ABS + polyurethane + PVC). Small surrogates (surrogate ID in Table 1 starts with “S”) were constructed with thinner sticks (1.91 cm thick) than the larger surrogates (surrogate ID starts with “L”; 3.81 cm thick). Sticks were arranged at three different packing densities (low, medium, and high) for small and large surrogates, creating six unique designs. Sticks were held together with wood glue (polyvinyl acetate, less than 0.5% by mass of any surrogate). No flame retardants were included in the surrogates.

Bryner and Mullholand defined a ventilation factor (ψ), calculated following eq 1, to quantitatively relate the surrogate design features (ratio of the open area of the vertical shafts of the surrogate, *A*_v_, to the total fuel surface area, *A*_s_) to smoke yields.

1 $$\psi =(b{h)}^{0.5}\left(\frac{A_{v}}{A_{s}}\right)$$

Equation 1 is based on an empirical observation that the surrogate’s burning rate per unit area is proportional to the stick thickness, *b*, and surrogate height, *h*.^23^ Despite the large variability in the total heat release across surrogate sizes and packing densities (e.g., 68 MJ for small low density surrogate and 500 MJ for large high density surrogate), the modified combustion efficiency (MCE = ΔCO_2_/(ΔCO_2_ + ΔCO)) integrated over each burn was between 0.9 and 0.95, indicating both well-ventilated flaming and smoldering combustion conditions as described by ISO19706.^24^

### Combustion Experiments

Combustion of the surrogates was performed under a 0.5 MW calorimeter at the National Fire Research Laboratory on the National Institute of Standards and Technology (NIST) campus in Gaithersburg, Maryland, in June of 2024. Operation of the calorimeter and smoke sampling is described in Bryant and Bundy.^25^ Experiments were conducted as follows: the mass flow of the hood exhaust was set between 2 to 3 kg s^–1^, the surrogate was placed on a digital scale under the calorimeter hood; small packages containing a high-molecular weight combustible fuel were placed on top of the surrogate (combustion of the ignition fuel made negligible contributions to NMOG emissions); a propane torch ignited the combustible packages to initiate the combustion of the surrogate; burning of the surrogates continued, lasting between 30 min (small low density) and 200 min (large high density); burning was extinguished after 55 to 65% of the initial mass was consumed. We calculated the mass expected at the end of each experiment as the ratio of dry noncombustible gypsum to the total mass of the wet surrogate to determine how much combustible mass was consumed. We found that the measured remaining mass at the end of each experiment (35% ± 3%) closely matched the remaining mass expected (38% ± 1%) providing evidence that the components of each surrogate were combusted completely. Each surrogate type (indicated by surrogate ID in Table 1) was burned in triplicate, totaling 18 burn experiments. Additional operational details and images of the surrogates are in Davis et al.

### Gas Sampling

Carbon monoxide (CO) and carbon dioxide (CO_2_) were measured with a nondispersive infrared gas analyzer (NDIR). Gases, including nitric oxide (NO), sulfur dioxide (SO_2_), methane (CH_4_), hydrogen cyanide (HCN), formaldehyde (HCHO), ethene, propene, acetylene, ethane, and 1,3-butadiene were measured using a Fourier transform infrared (FTIR) gas analyzer.

NMOGs were measured using hydronium ion (H_3_O^+^) proton-transfer-reaction time-of-flight mass spectrometry (PTR-MS). Our PTR-MS instrument settings and calibration methods, including the parametrization of NMOG sensitivity versus proton-transfer rate constant (k_PTR_), have been described previously.^26^ We used a gas-chromatograph interfaced with the PTR-MS to characterize isobaric ion interferences for two surrogate burns to inform our identification of ion signals.^27^ We report mass-to-charge ratio (m/Q) in units of Thomson (Th) which is equal to 1.0364 × 10^–8^ kg C^1–^.

We quantified 201 ion signals from PTR-MS measurements following the criteria that they were largely free of undesirable product ion interferences (e.g., fragmentation/water cluster product ions) and were enhanced above background during combustion experiments. We directly calibrated for more than 40 NMOGs. NMOGs for which we were confident of their identity, but did not have a calibration for, we either calculated sensitivities using the sensitivity versus proton-transfer rate coefficient (k_PTR_, calculated from the parametrization of Sekimoto et al. using dipole moments and polarizabilities),^26^ used a sensitivity (units of ion counts per second per parts-per-billion, cps per nmol mol^–1^) of a structurally similar NMOG, or assigned a sensitivity value of 2000 cps per nmol mol^–1^ for ions that could not be confidently assigned identities or likely structures. We measured NMOGs containing chlorine that have not been reported in combustion measurements elsewhere. Many chlorine-containing NMOGs and NMOGs with mass-to-charge ratios above 150 Th were assigned a sensitivity of 2000 cps per nmol mol^–1^ which is the average sensitivity measured for several chlorine-containing NMOGs with direct calibrations. Future measurements of chlorine-containing NMOGs using an analytical technique like thermal desorption gas-chromatography mass spectrometry could aid in identification of NMOGs measured by the PTR-MS and improve quantification of yields. More information on NMOG identification and quantification is in the Supporting Spreadsheet.

A 10 m, 9.5 mm outer diameter polyethylene sample line pulled air from the hood exhaust duct at 5 L per minute (Lpm). Air was subsampled from the polyethylene sample line at 4 Lpm through 1 m of 6.35 mm PFA tubing and the PTR-MS subsampled 120 mL per minute from that flow. A 10 μm PTFE filter was placed between the polyethylene and PFA sampling lines to filter particles from the PTR-MS sample air. The filter was replaced after every burn. PTR-MS sample air was diluted by a factor of 3.5 with ultrapure zero air at the inlet to minimize reagent ion titration effects. The residence time of sample gas from the combustion site to the instruments was approximately 10 s for the FTIR and NDIR and 15 s for the PTR-MS. We did not heat the PTR-MS sample line and thus reported yields for semi volatile species (e.g., naphthalene, C_11_, and C_12_ aromatics) may be lower bounds.

### Data Processing and Yield Calculations

All gas data from the three instruments were collected at a 1 Hz sample rate and a 10 s running average was applied for final analysis. Background gas concentrations measured 1 min before the start of each burn experiment were averaged and subtracted from the gas time series measured during each surrogate burn. Three combustion experiments were performed for each type of surrogate except for large medium and large high densities, where only one burn was sampled.

We quantified yields (Y, g_gas_ kg_fuel,dry_^–1^) of smoke constituents from constituent emission rates (*E*_r_, g s^–1^) following eqs 2 and 3

2 $$E_{r,X}=\left[X\right]\left(\frac{MW_{X}}{MW_{\mathrm{air}}}\right)ṁ$$

3 $$Y_{X}=\frac{\int E_{r,X}\mathrm{dt}}{\Delta M_{\mathrm{wet}}}\left(\frac{M_{\mathrm{wet}}}{M_{\mathrm{dry}}}\right)$$

where [*X*] is the background-subtracted mole fraction of a smoke constituent in air (mol mol^–1^), MW is the molecular weight of a smoke constituent, *X*, or air (g mol^–1^), *ṁ* is the hood exhaust mass flow rate (kg s^–1^), *M*_wet_ and *M*_dry_ are the total and dry masses lost (kg) from the combusted surrogate. To determine dry mass, a surrogate from every size and packing density classification was placed in an oven for 24 h at 105 °C then transferred to a desiccant for 5 h before weighing. We used a single value of 1.29 for $\left(\frac{M_{\mathrm{wet}}}{M_{\mathrm{dry}}}\right)$ determined from measurements of material water content presented in Davis et al. All the yields reported in this manuscript are mass of NMOG per mass of dry fuel consumed. Integration of smoke constituent mole fractions was started at ignition and terminated at the end of the sampling period (defined as the time when the surrogate was extinguished). All uncertainties are reported with a confidence level of 2σ unless otherwise noted.

### NMOG Time Series Analysis using Positive Matrix Factorization (PMF)

We analyzed NMOG time series measured by the PTR-MS (NMOG_PTR_) using positive matrix factorization (PMF). NMOGs measured by the FTIR (i.e., ethane, ethene, pentane, etc.) were not analyzed with PMF so they could be used to compare with the NMOG PMF factors determined from PTR-MS measurements. When applied to mass spectral data, PMF is a non-negative numerical method that reconstructs time series of hundreds of ion signals using the product of factor time series and factor mass spectral contributions.^28,29^ Users define the number of factors, and PMF calculates time series and mass spectral profiles for those factors. PMF factors are typically used to understand sources of NMOGs. For our study, factors produced from PMF analysis may represent NMOG sources either directly emitted from the surrogates and/or produced from combustion chemistry. Details of the PMF algorithms and formulations can be found elsewhere.^30,31^

We performed PMF on each burn experiment using the PMF Evaluation Tool v.3.08E for analyses and solution optimization.^28^ NMOG_PTR_ mole fraction data matrices for the 201 quantified ions were used as inputs to PMF. Error matrices were generated by dividing the statistical ion counting uncertainties in units of ion counts per second to nmol mol^–1^ using NMOG_PTR_ sensitivities. Improvements in factor validation (i.e., factor time series correlations with gases measured by the FTIR) were observed after applying a scaling factor of 9 to the entire error matrix. Benzene mole fractions were typically higher than other NMOGs, so we also applied a factor of 10 scalar to the error for benzene (a procedure also known as “downweighting”). We show a sensitivity analysis to the choice of the error matrix in the Supporting Information and find that, although the objective function could vary over 2 orders of magnitude, our three-factor PMF mass spectral profiles and time series were insensitive to the treatment of the error matrix across the range we tested (Figure S2, Tables S1 and S2). Ultimately, we rely on time series correlations of our PMF factors with gases measured by the FTIR and relating NMOG composition of factors to combustion of known components in the mixed fuel surrogate to support our identification of PMF factors. Additional details of the PMF analyses are in the Supporting Information.

## Results and Discussion

### Gaseous Emissions from Building Surrogate Combustion

Figure 1 shows smoke constituent yields (Y) for NO, SO_2_, and CH_4_, as measured by the FTIR (Figure 1A through 1C), and NMOGs (Figure 1D through 1J), as measured by the PTR-MS.

**Figure 1:**
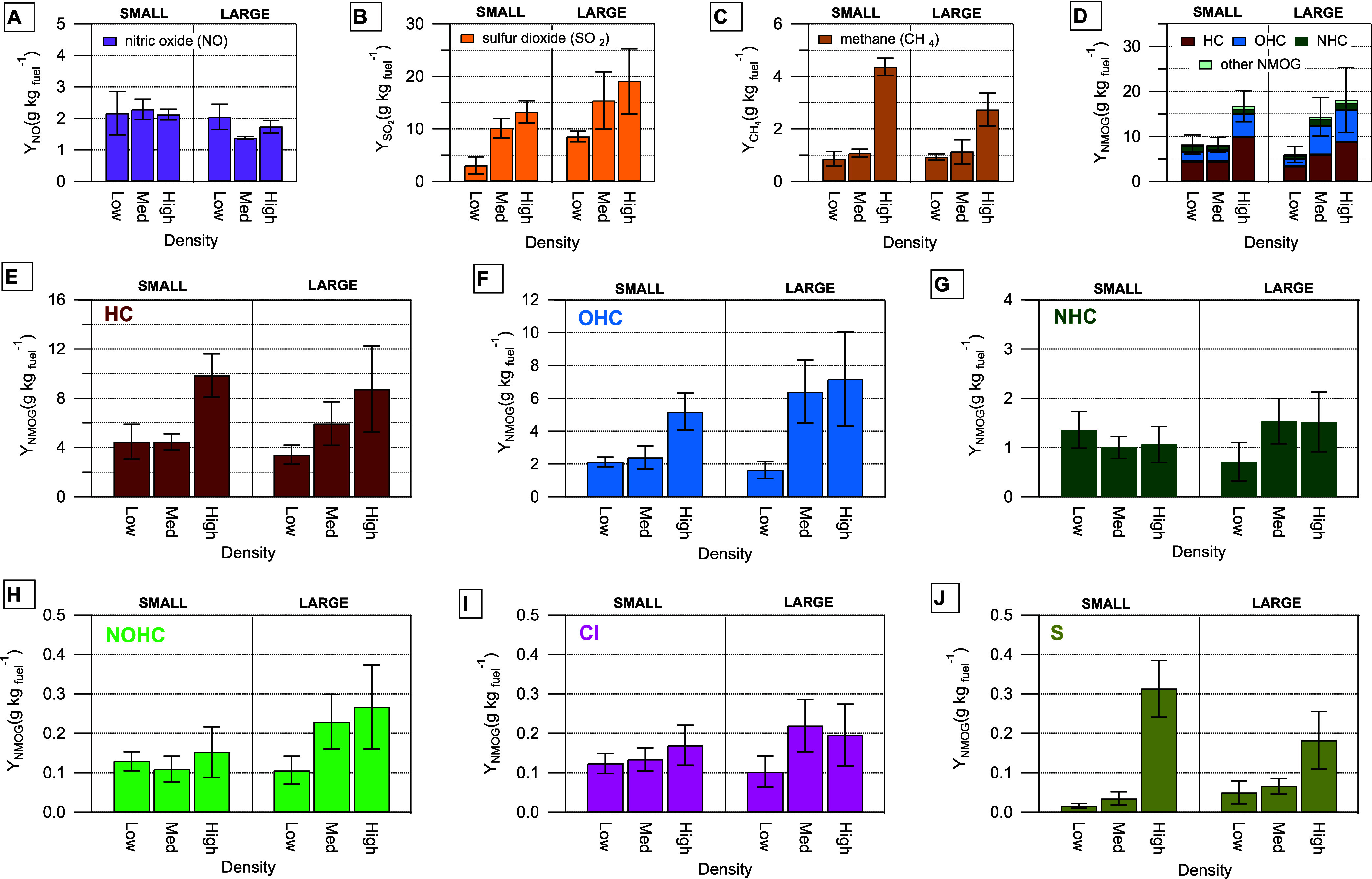
Gas yields (Y, g of gas per kg of dry fuel) of (A) NO, (B) SO_2_, (C) CH_4_ as measured by the FTIR, and (D) summed nonmethane organic gases (NMOG) as measured by both the FTIR (for select gases) and PTR-MS. NMOGs are further categorized according to elemental composition: (E) HC, gases containing only carbon and hydrogen, (F) OHC, oxidized HC, (G) NHC, nitrogen-containing HC, (H) NOHC, nitrogen and oxygen HC, (I) Cl, chlorine-containing HC, and (J) S, sulfur-containing HC. “Other NMOG” in Panel D are the sum of NMOGs in panel H–J. Yields are shown as a function of surrogate size (small and large) and density. Relative standard deviations of 30 and 40% were applied to the yields for the large medium density and large high density surrogates (estimated from corresponding organic gas yields determined from triplicate FTIR measurements). All other error bars show the standard deviation of three replicate measurements. Figure S1 shows these data along with individual NMOG contributions to chemical classes in E–J.

We use NO, SO_2_, and CH_4_ as case examples to demonstrate how variation in combustion processes as a function of surrogate size and stick packing density may or may not contribute to differences in yields. Surrogate size and packing density are related to ventilation conditions as previously described in eq 1.

Figure 1A shows the Y_NO_ to demonstrate how consistent well-ventilated high-temperature combustion of nitrogen-containing fuels was between surrogates. NO is generated from flaming combustion of fuel containing nitrogen, when temperatures are typically between 800 and 1100 °C.^32^ Based on the nitrogen fuel content by mass, most fuel-based nitrogen comes from ABS (est. nitrogen content of 6% by mass^33^) and polyurethane rubber (est. 5 to 8% nitrogen by mass^34^). The Y_NO_ for a given surrogate is within 20% of the average of all surrogates indicating the fuel responsible for NO generation underwent a consistent amount of well-ventilated flaming combustion within the range of surrogate sizes or densities tested here.

In contrast to NO, Y_SO2_ increased as both surrogate size and density increased (Figure 1B). SO_2_ is produced from the breakdown of gypsum (CaSO_4_ · 2H_2_O) after heating above 230 °C.^23^ Although the mass fraction of gypsum in the surrogate was constant across all surrogates, the Y_SO2_ increases as the total mass of gypsum increases. Thus, SO_2_ emission will occur from the gypsum as long as the surrogate is heated sufficiently long to evaporate chemically bound water which initiates conversion of mineralized sulfur to SO_2_.^8^

We expect increased Y_CH4_ to indicate incomplete combustion when the availability of oxygen is limited. Only the highest density surrogate configurations (small high and large high density), which may have had limited oxygen for combustion, have Y_CH4_ that are larger than the measured range (the average and standard deviation) from the other surrogates (Figure 1C).

We categorize the 201 NMOGs quantified in this study into six categories based on their elemental composition: hydrocarbons (HC, contain only carbon and hydrogen), oxygenated HC (OHC), nitrogen-containing HC (NHC), nitrogen and oxygen containing HC (NOHC), chlorine containing HC (Cl), and sulfur containing HC (S) (Figure 1E through 1J). Average Y_NMOG_ composition across all surrogates is 52% ± 7% HC, 33% ± 8% OHC, 11% ± 4% NHC, and 4% ± 1% for all other NMOGs (Figure 1D). Increases in total Y_NMOG_ are driven by increases in HC and OHC compounds that increase with increasing surrogate density and size. Yields of NOHC, Cl, and S compounds were much lower (approximately an order of magnitude or more) compared to the other three categories. Although we do not have measurements of HCl or Cl_2_, the relatively low yields of Cl compounds may suggest that combustion of PVC (the only appreciable source of chlorine in this study) is mostly generating Cl-containing end products (HCl and Cl_2_) as opposed to other incomplete combustion products. Comparisons of select NMOG yields from the surrogates to yields measured from room/building-scale^4,22^ and biomass^35^ fires are presented later in the manuscript.

### Factor Analysis of NMOG_PTR_ Time Series Shows Surrogate Component Combustion

From our PMF analyses of NMOG_PTR_ time series, we find that a three-factor solution best recreates the total NMOG_PTR_ mole fraction time series from the surrogate burns while explaining key sources of NMOG_PTR_ during combustion. We explored solution spaces up to seven factors and found that combustion processes that made minor contributions to NMOG_PTR_ yields could be discerned as more factors were included in the PMF results (Figures S3, S4, and S5), but interpretation of the factors became more complicated and less generalizable across surrogates (additional details in Supporting Information). Figure 2 shows an example of PMF results for a three-factor solution from the combustion of a small medium density surrogate.

**Figure 2:**
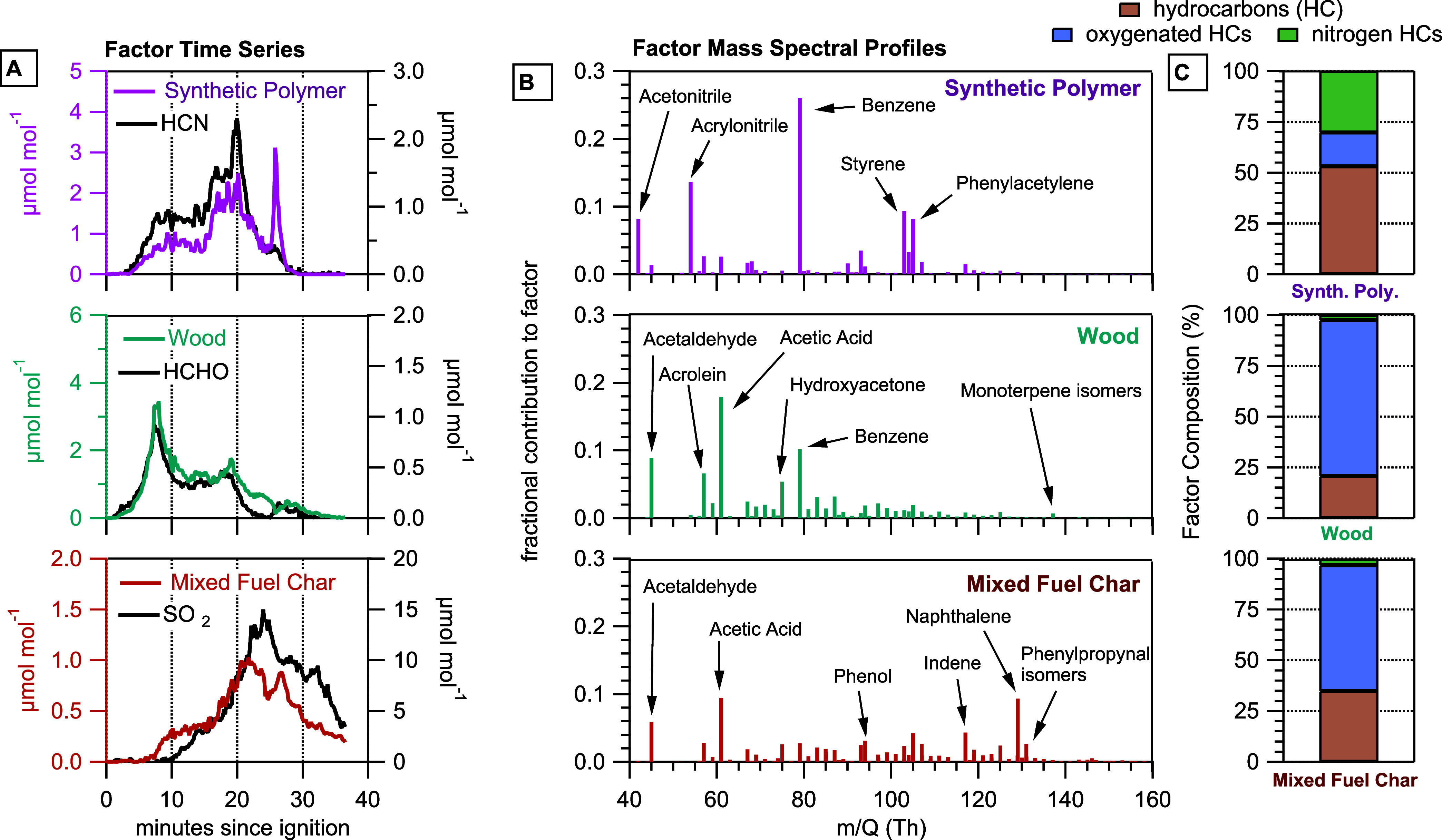
Example factor analysis results for a small medium density surrogate. (A) Mole fractions of the three different NMOG_PTR_ factors, synthetic polymers, wood, and mixed fuel char, plotted with tracer species measured by the FTIR. Hydrogen cyanide (HCN) is the tracer for synthetic polymers, formaldehyde (HCHO) is the tracer for wood, and SO_2_ is the tracer for mixed fuel char from this surrogate. (B) Mass spectral profiles corresponding to the factors highlighting select NMOGs. (C) Percent composition (μmol_NMOG,i_/∑μmol_NMOG_) of the factors categorized by chemical class.

We identified three factors corresponding to the combustion of synthetic polymers (ABS and polyurethane), wood, and mixed fuel char. Several lines of evidence including video footage of the fire, factor mass spectral profile correlations, and time series correlations with gases measured by the FTIR were used to support the identification of the factors. We use the factors determined from this analysis to connect how the chemical composition of NMOG_PTR_ is changing as a function of time during the combustion experiments and how that likely relates to combustion conditions.

The synthetic polymer factor time series correlated with hydrogen cyanide (HCN, Figure 2A and other correlations in Figure S6) and was composed of NMOGs expected to come from the combustion of nitrogen-rich ABS and polyurethane, such as nitriles (e.g., acrylonitrile, acetonitrile, benzonitrile) and reduced aromatics (e.g., benzene, styrene, phenylacetylene) (Figure 2B). We expect polyurethane and ABS to be important sources of reduced aromatics like benzene because the monomeric units of those polymers are connected by aromatic rings. The relative contributions of NMOGs to the mass spectral profiles determined for each surrogate were consistent and profiles correlated well (r^2^ > 0.8) between surrogates (Figure S7). Although PVC is a synthetic polymer, we do not see obvious tracers for PVC combustion, such as vinyl chloride, correlating with this factor profile. NHCs were always >25% of the molar composition for this factor compared to the relatively low (i.e., <1%) NHC composition of the wood and mixed fuel char factors (Figure 2C).

The wood factor time series correlated with formaldehyde (HCHO, Figure 2A and other correlations in Figure S8) and had OHC species that made important contributions to the factor profile, like acetaldehyde, acrolein, acetic acid, and hydroxyacetone (Figure 2B). In the initial phase of biomass combustion, distillation of highly volatile vapors (like terpenoids) occurs and acts as a source of NMOGs (monoterpene isomers can be seen in the wood factor profile in Figure 2B). As biomass is further heated, depolymerization of cellulose, hemicellulose, and lignin create volatile carbon and oxygen-rich monomers that evaporate and become sources of NMOGs. Thus, we expect a major source of small volatile OHC compounds in the wood factor to originate from biopolymer (cellulose, hemicellulose, and lignin) pyrolysis. In our study, benzene associated with the wood factor could come from both combustion of the phenolic resin in the OSB and aromatization of biopolymers in both woods (SPF and OSB).

The mass spectral profiles for the wood factor were consistent between surrogates (r^2^ > 0.75), but they also correlated well (r^2^ > 0.80) with the “high temperature” and “low temperature” pyrolysis NMOG_PTR_ PMF factors measured from the biomass burning experiments described in Sekimoto et al. (Figure S9). In contrast to this study, the combustion experiments highlighted in both the studies of Sekimoto et al. and Roberts et al. did not include mixed fuel packages and thus were able to differentiate between high and low temperature pyrolysis of biomass. Thus, those two studies could identify NMOGs originating from high and low temperature combustion whereas the role of temperature on combustion of individual materials in our study was not obvious. Our PMF analysis could not differentiate high and low temperature pyrolysis, and we hypothesize the wood factor from our study represents emissions from a combination of both high and low temperature combustion of both SPF and OSB woods.

Eventually biomass is reduced to char which can emit volatiles like benzene and naphthalene formed from the aromatization of biopolymers (Figure 2B).^29^ Compared to biopolymers, the synthetic polymers combusted here are likely to generate less complicated emission profiles due to the breakdown of simpler polymer chains that comprise the material. The time series of the mixed fuel char factor correlated with SO_2_ (Figure 2A) for all the surrogates except for the large medium density, which correlated with water vapor (Figure S10). The mixed fuel char mass spectral profile generally did not correlate well (r^2^ < 0.5 for all surrogates) across the data set indicating the composition of this factor was more variable than the other two. When expanding the PMF results to a four-factor solution this factor would typically mathematically split to form another factor that was largely defined by a single NMOG like benzene, acetic acid, or naphthalene (Figure S11). We hypothesize that the NMOGs representing the mixed fuel char factor originate from a combination of charred wood, synthetic polymers, and thermal degradation of the gypsum possibly leading to an inconsistent chemical composition across surrogates.

PMF factors were typically observed at the same stage of a given experiment, with the wood factor appearing at the beginning, the synthetic polymer appearing next, and then the mixed fuel char factor (Figure 3). We observe that combustion of individual components in the mixed fuel surrogate are likely driving changes in the heat release rate (HRR) and regulating the production of NMOGs. For lower density surrogates (small and large low density, Figure 3 SL and LL), combustion is well-ventilated and structures are heated near-homogeneously resulting in near-sequential rapid combustion of wood and then synthetic polymers to form mixed fuel char. The wood factor typically appeared when dHRR was positive, indicating that exothermic combustion of wood volatiles was driving the increase in HRR and the surrogate’s temperature. Wood ignites at lower temperatures (autoignition temperature range of 200 to 300 °C for SPF/OSB^36,37^ ) than the nonwood components of the surrogates (autoignition temperature range of 310 to 560 °C for polyurethane/ABS/PVC^38–40^ ) and thus may explain why we typically see the wood factor appearing before the synthetic polymer factor. We hypothesize that the initial breakdown of wood biopolymers drives the increase in NMOGs from this factor. With increasing surrogate size and density (corresponding to decreasing air ventilation), increases in NMOG mole fractions are largely a result of emissions of unburned NMOGs. For the denser surrogates, heat distribution is heterogeneous (most pronounced with small and large high density, Figure 3 SH and LH) and thus NMOG_PTR_ factors appear simultaneously as wood and synthetic polymers combust (and char emits) gradually during the experiment. For the low and medium density surrogates after the peak HRR has occurred, combustion starts to become less efficient and fuel nitrogen starts combusting to produce NMOG_PTR_ species like acrylonitrile and acetonitrile, which strongly contribute to the synthetic polymer factor, instead of end-products like NO (Figure S12).

**Figure 3:**
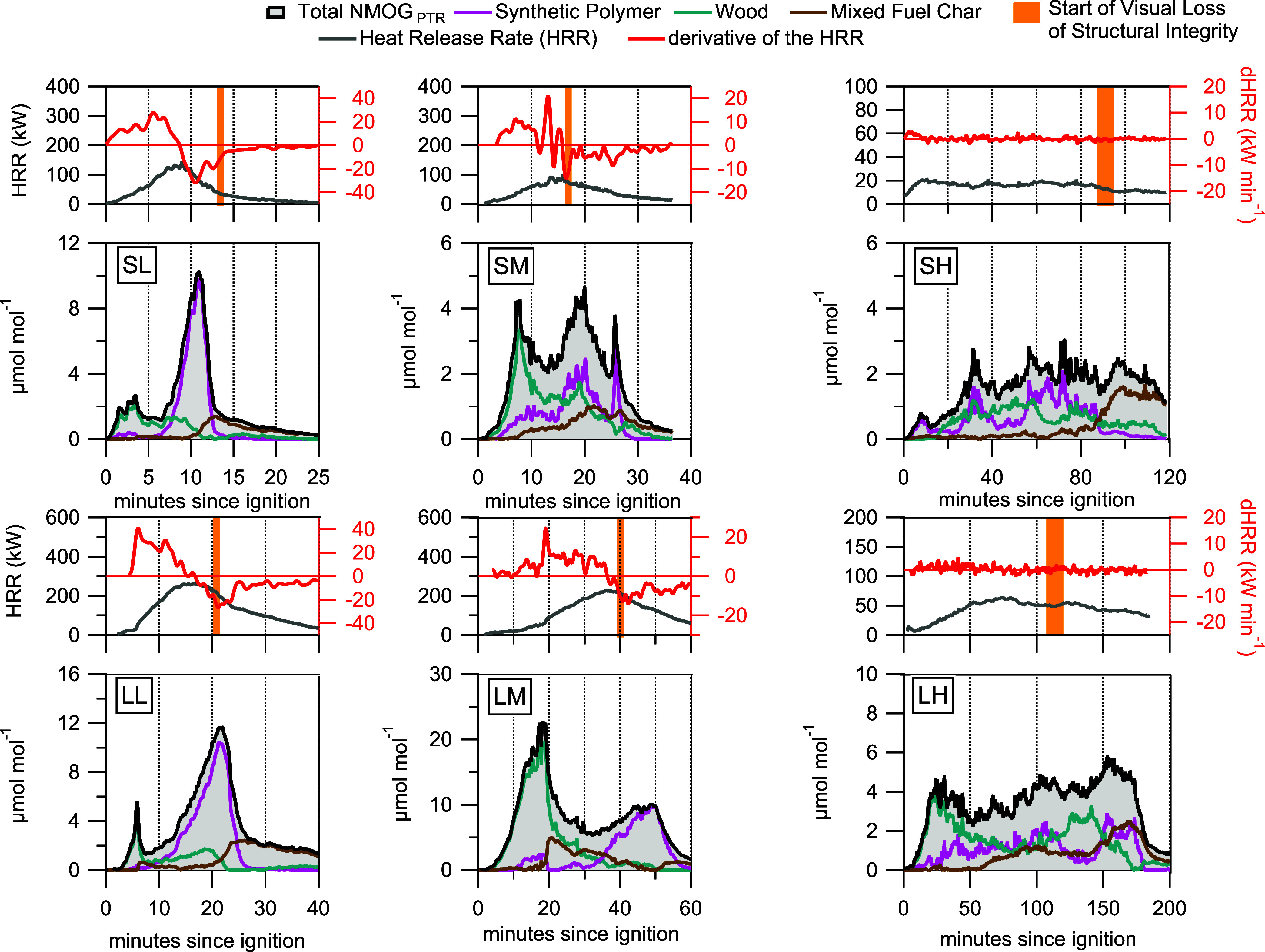
Time series of NMOG_PTR_ factors plotted with the heat release rate (HRR, right axis of upper panels) and first derivative of the HRR (dHRR). Surrogate identity (S = small, L = large size and L = low, M = medium, and H = high density) is shown in the NMOG time series panel and the corresponding time series for HHR and dHRR is plotted directly above. An orange shaded area on the upper panels shows the approximate time a loss of structural integrity could be observed in recorded video of the surrogate burns likely indicating the formation of char.

Shortly after the wood factor mole fraction peaks, the dHRR starts to decrease possibly from the combined effects of decreasing wood fuel availability and the endothermic phase transition of the synthetic polymers to semisolids and liquid (Figure 3 SL, SM, LL, and LM). Combustion of the synthetic polymers in our surrogates first occurs through melting, thermal breakdown of the semisolid material, and then the creation of char after passing through a glass transition (if sufficiently cooled). Pools of melted ABS can be seen in the base of the surrogate in Figure S13. The steepest decrease in the HRR (most negative values of dHRR) typically occurs when the synthetic polymer factor is at peak mole fractions. The dHRR starts to increase shortly after the peak in synthetic polymer factor mole fraction likely indicating the completion of polymer fuel consumption. For the highest density surrogates (Figure 3 SH and LH), as oxygen availability to the entire fuel package is limited and combustion proceeds slowly (i.e., low peak HRR) there is no clear relationship between HRR and the appearance of the NMOG_PTR_ factors. Thus, factors appear nearly simultaneously as upper layers of the fuel package combust faster than the lower layers.

Based on time series analysis, the mixed fuel char factor typically appeared toward the end of a burn and when signs of glowing smoldering combustion could be seen in the visual evidence of the burn (Figure S13). In Figure 3 we show orange shaded areas to indicate the approximate time during the burn when loss of structural integrity can be seen in the video recording. We hypothesize this loss of structural integrity is related to the formation of mixed fuel char from pyrolyzed fuel. When the mixed fuel char factor increases, it coincides with when most of the combustible fuel is burned and gypsum comprises most of the remaining material in the glowing stage, therefore increasing the SO_2_ concentration (Figure S10). The inconsistent NMOG composition of the char factor suggests that incomplete combustion of surrogate component materials may produce variable mixed fuel char that may off-gas different NMOGs (like acetic acid and naphthalene) as it undergoes oxidative pyrolysis at the end of the combustion experiment.

PMF factors were not associated with clear patterns of increasing or decreasing MCE (Figure S12). The mixed fuel char factor was at the highest concentration, for both the small and large high density surrogates (Figure S12, SH and LH), when the MCE was between 0.8 and 0.9, but was at high concentrations when MCE was approximately 0.95 for the other surrogates. The appearance of the wood factor in the large surrogates (Figure S12, LL, LM, and LH) coincided with a pronounced dip in MCE (e.g., from 0.95 to between 0.8 to 0.9), but this was not observed in the small surrogates.

### NMOG_PTR_ Yields Increase with Decreasing Ventilation Factor

We calculated PMF factor yields by calculating yields of NMOGs contributing to a given factor, then summing all the NMOG yields in the factor profile (Y_NMOG, PTR total_ = ∑Y_NMOG, PTR PMF profiles_). Figure 4A shows that total NMOG_PTR_ yields from the surrogates follow the ventilation factor and can be fit to a power law relationship, with higher NMOG_PTR_ yields occurring at lower ventilation factors. Although useful for differentiating the contributions to total NMOG yields from the different materials in the mixed fuel surrogates, the power law fits shown in Figure 4 are dependent on yields of NMOGs specific to the PTR-MS used in this study and the ventilation factor defined by the design of the surrogate and thus may have limited applicability to other studies.

**Figure 4:**
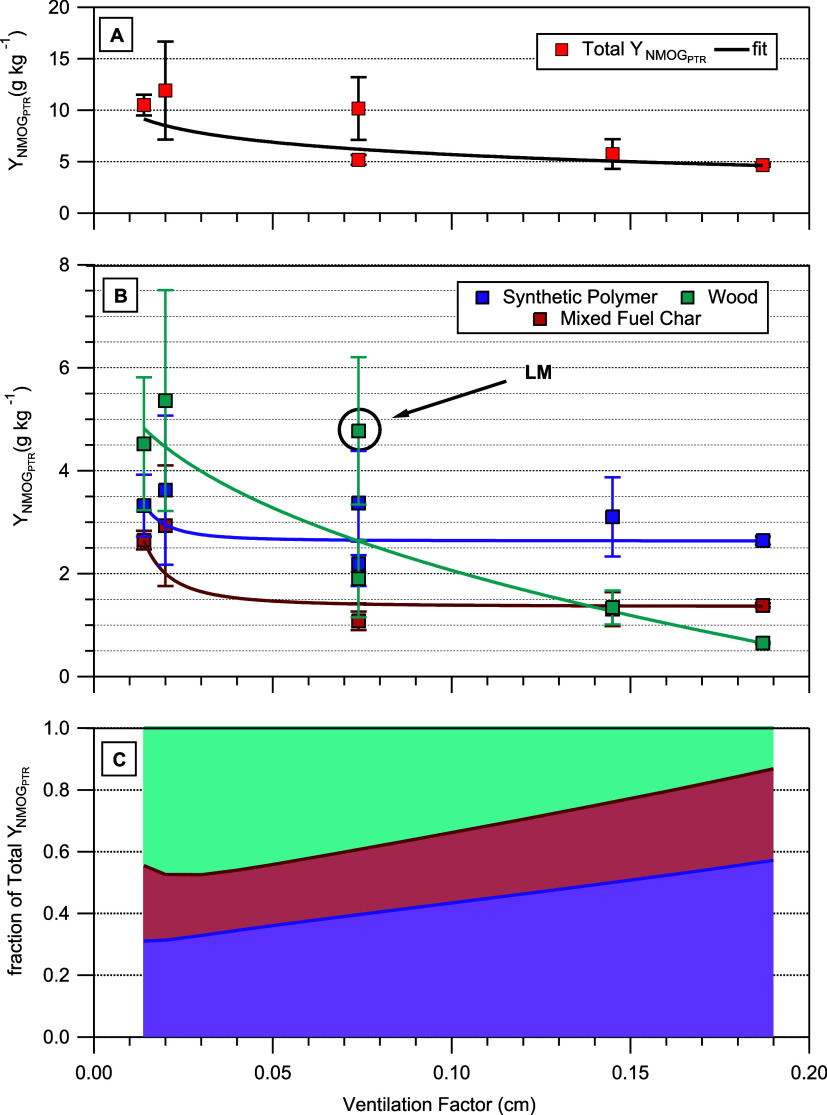
(A) The total yields of NMOG_PTR_ as a function of the ventilation factor. Lower ventilation factors correspond to denser surrogates. A weighted power law fit to the data is shown. (B) Factor yields as a function of the ventilation factor. Power law fits, weighted by the standard deviation (2σ), were performed on the yields from each factor. A relative standard deviation of 30% and 40% was applied to the LM and LH surrogate yields. (C) The fraction of the total Y_NMOG,PTR_ attributable to each factor as a function of the ventilation factor. Fractional contributions were determined by calculating Y_NMOG,PTR_ for each factor from the power law fits shown in (B) and dividing by the total Y_NMOG,PTR_.

The main driver of increased total Y_NMOG,PTR_ as the ventilation factor decreases (as shown in Figure 4A) is the increase in the contribution of the wood factor to Y_NMOG,PTR_ as shown in Figure 4B. The Y_NMOG,PTR_ from the wood factor increases from 0.65 to 4.52 g kg^–1^ (factor of 7 increase) going from the highest ventilation factor to the lowest. In contrast, the yields of the other two factors stay approximately constant at all ventilation factors with only smaller (<factor of 2) increases in yields at the two lowest ventilation factors. Figure 4C demonstrates that, at the highest ventilation factor, synthetic polymer combustion accounts for approximately 60% of the total Y_NMOG,PTR_ which decreases to approximately 30% at the lowest tested ventilation factor. This result demonstrates that NMOG emissions from fires of structural wood components may be most sensitive to reduced oxygen combustion conditions.

### NMOG_PTR_ from Surrogates and Comparisons to Structure and Biomass Fires

Many of the NMOGs generated from combustion of the surrogates have been measured from biomass^3,35,41–43^ but limited measurements exist from structural fires on the building-scale. Figure 5 shows comparisons of select NMOG yields, allocated by PMF factor source contribution, separated by chemical category and compared to literature reports from biomass and structural fires (when available). We show NMOG yields from the small low density and large high density surrogates to represent approximate lower and upper bounds of observed yields from our experiments. The values corresponding to the room/building-scale fires in Figure 5 come from the emissions inventory of Holder et al. filtered for experiment scales limited to room and full building fires. Biomass comparison values are taken from the Smoke Emissions Reference Application (SERA) database which includes measurements collected from the combustion of many different biomass types with different combustion efficiencies from field and laboratory studies.^35^ We did not apply any filters to the SERA database based off of combustion conditions (e.g., flaming, smoldering), biomass type (e.g., grasses, trees), or whether the data were collected from an aircraft or in a laboratory experiment. We limit our comparison of NMOG yields to those reported from structure fires and biomass to highlight any possible differences or similarities in NMOG emissions that could be expected from WUI structure fires versus wildfires in North America.

**Figure 5:**
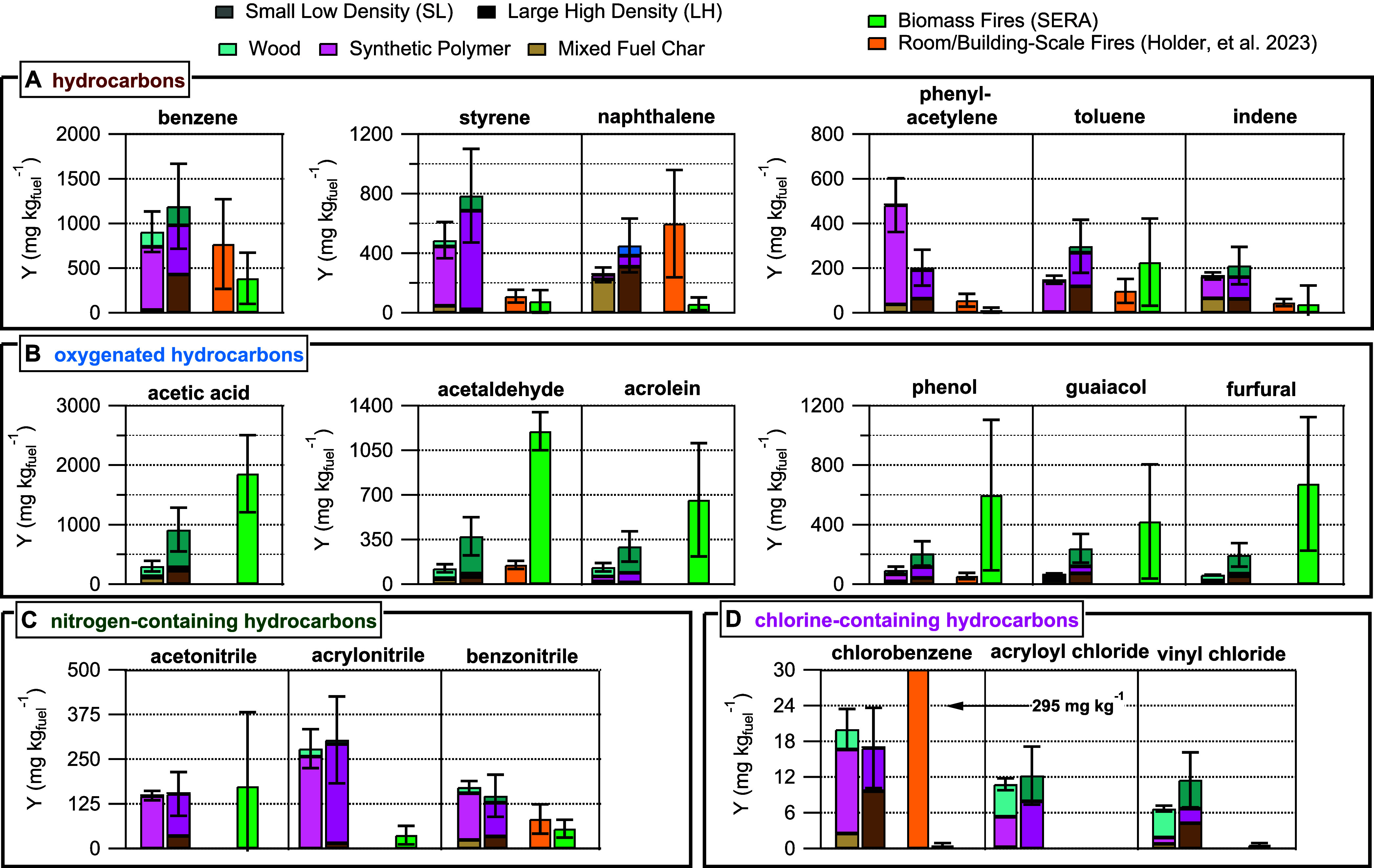
Select NMOG_PTR_ yields separated by chemical classification, (A) HC, (B) OHC, (C) NHC, and (D) Cl, and compared to yields reported from previous studies for room/building-scale and biomass fires. The height of the bar shows the total yield for a given NMOG, where the small low density surrogate (lighter colors) is on the left and large high density surrogate (darker colors) is on the right. Colored portions of the bar correspond to fractions of the yield attributable to one of the three PMF factors. Error bars show the SL surrogate’s standard deviation of triplicate yield values and the estimated 40% error for the LH surrogate. Room/Building-scale fire yields (orange) are taken from the database compiled by Holder et al., and the biomass fire yields are taken from the Smoke Emissions Reference Application database (SERA, green). Error bars for the literature values show the standard deviation of available yields. A blank spot where a biomass or room/building value should be indicates that a comparison value was unavailable. The room/building-scale comparison value for chlorobenzene is high compared to surrogate measurements, so the figure shows the yield value.

HC compound yields measured from the surrogate burns show variable agreement with biomass and structural yield values (Figure 5A). Total yields of styrene and phenylacetylene are notably higher than the room/building-scale yields possibly pointing to the importance of ABS and polyurethane combustion as a source of NMOGs from our surrogates compared to other studies. The contribution of the yields attributable to the wood factor for HC compounds in Figure 5A (except toluene and naphthalene) fall within the standard deviation of average values reported from the SERA database for biomass. For instance, Figure 5A (panel corresponding to benzene) the contribution of the wood factor to the benzene yield for the average of the low and high density surrogates (190 ± 30 mg kg^–1^) falls within the range of what is reported for biomass from the SERA database (380 ± 290 mg kg^–1^).

Compared to biomass, OHC compounds generated from the surrogates show lower yields but fall within the range of what has been reported for room/building fire yields for acetaldehyde and phenol (Figure 5B). We expect many of these OHC compounds to be generated from biomass combustion and accordingly Figure 5B shows >50% of several OHC compound yields (acetic acid, acetaldehyde, acrolein, furfural) are attributable to the wood factor. Lower yields of OHC compounds from the surrogates are in part explained by considering 70% of the total combustible mass of mixed fuel was wood thus creating low yields compared to pure biomass fuel (as represented by the SERA comparison values).

Synthetic polymer combustion was the primary source for all NHC compounds measured in our experiments. Interestingly, the agreement of the acetonitrile yield from our surrogates with biomass shown in Figure 5C reflects a similar yield from two different types of fuels. The only source of chlorine-containing NMOGs was PVC and literature reports of smoke yields are scarce. Notably, the large disagreement between the high yield of chlorobenzene from a single structure fire compared to the surrogates is likely because the structure contained a sofa.^22^ Chlorobenzene yields from PVC combustion have also been reported to be relatively low and could explain why chlorobenzene yields from our surrogates are low compared to the room/building fires.^44^ In addition to direct emissions from combustion, we note that chlorine radical reactions with coemitted hydrocarbons from wood may affect the variety and abundance of Cl NMOGs from our surrogates^45^ and possibly produce species like acryloyl chloride.

## Implications

Homes nearby an area affected by WUI fires may be contaminated with smoke from burned structures. Such smoke contamination may impact indoor air quality. Several of the NMOGs identified as making major contributions to PMF factor yields here may be possible candidates for WUI structure fire tracer species in indoor air. For example, synthetic polymer combustion was a more important source of reduced cyclic aromatics, like benzene and styrene, than the wood and mixed fuel char factors combined. Synthetic polymer combustion also produced yields of nitriles that were an order of magnitude higher than wood from our surrogates. However, both reduced aromatics and nitriles can be present at elevated concentrations (e.g., 0.5 to 1 nmol mol^–1^) indoors^46^ possibly complicating their utility as WUI smoke contamination tracers. In one example, Dresser et al. had difficulty differentiating the exact contributions of native indoor emission sources versus WUI smoke contamination to gas-phase concentrations of reduced aromatics in a home contaminated by smoke from the Marshall Fire in Colorado. The authors measured elevated concentrations of NMOGs likely associated with the WUI fire event indoors that decayed over several weeks but had to use factor analysis to differentiate background from smoke contamination sources. No one chemical or set of chemicals measured from this study is expected to serve as a reliable tracer on its own because of non-WUI sources from both outdoor air and emissions from the indoors. However, the PMF factors identified in this study may provide examples of chemicals (e.g., reduced aromatics, nitriles) that may be elevated in indoor air in homes contaminated by WUI fire smoke.

Factor analysis showed that the composition of our building surrogates will largely drive the smoke composition, and surrogate design (size/stick packing density) will affect smoke composition to a lesser extent. We observed that decreasing surrogate ventilation increased the yield of NMOGs from wood by a factor of 4 (going from the highest to the lowest ventilation). Similarly, the total NMOG yield (from the sum of wood, synthetic polymers, and char) increased by a factor of 2. For comparison, yields reported in the SERA database for benzene varied over an order of magnitude for different biomass fuel types and thus the effects of ventilation on NMOG yields observed here might be considered modest in comparison to variability across biomass fuels. The surrogates evaluated in this study provide an opportunity to examine the emissions from complex mixed fuels, possibly encountered in larger structures, by including component materials like carpet, wiring, or insulation. Additionally, these surrogates are more time and cost-effective to burn than a full structure.

Few measurements of NMOG yields from residential building materials exist and here we provide such data to aid in the understanding of likely NMOG smoke constituents from residential building fires at the WUI. A robust determination of how representative the NMOG yields measured from the surrogates in this study are compared to room and building-scale fires is limited in part because few studies have reported emissions of NMOGs from full-scale experiments. We show that the residential building surrogates in this study could be a useful tool for generating smoke from mixed fuel packages for the study of structure fires. However, future measurements of building-scale fires are warranted to further quantify yields of NMOGs, measured from smaller-scale experiments (like the surrogate burns described here), that have known or suspected toxicity, such as acrylonitrile, acrolein, and acryloyl chloride, and have not been reported from existing building-scale fire studies. Additionally, information on the combustible mass of residential buildings, typical of the housing stock in the United States is limited.^47^ Such information would be informative for constructing fuel packages with component mass fractions representative of different parts of the existing housing stock located in geographically diverse WUI areas.

## Supplementary Material







## Notes

Detailed information on the PMF analysis method including limited results from the seven-factor solution, additional trace gas time series, and NMOG yields (PDF)
Descriptions of the 201 PTR-MS ion identities and sensitivities (XLSX)
Information about NMOG individual yields and PMF factor yields as well as additional PMF factor profile data (XLSX)
