# Supplementary material for: Wildland-Urban Interface (WUI) Smoke Yields of Nonmethane Organic Gases from Combustion of Small-Scale Residential Building Surrogates
Source: ACS EST Air. 2025 Oct 9;2(11):2455–66. doi: 10.1021/acsestair.5c00187 (PMC12624524; doi:10.1021/acsestair.5c00187)
Supplement: Supplementary file 1 [file ea5c00187_si_001.pdf]

Supplemental Information for:

Wildland-Urban Interface (WUI) Smoke Yields of Non-Methane Organic Gases from Combustion of Small-Scale Residential Building Surrogates

Michael F. Link, Aika Y. Davis, Nathan M. Lima, Ryan L. Falkenstein-Smith, Rodney A. Bryant, Thomas G. Cleary, Dustin Poppendieck*

National Institute of Standards and Technology, Gaithersburg 20899 Maryland, United States

**Corresponding author email: michael.f.link@nist.gov*

Number of Pages: 21

Number of Figures: 14

Detailed Figure 1 from Main Text.

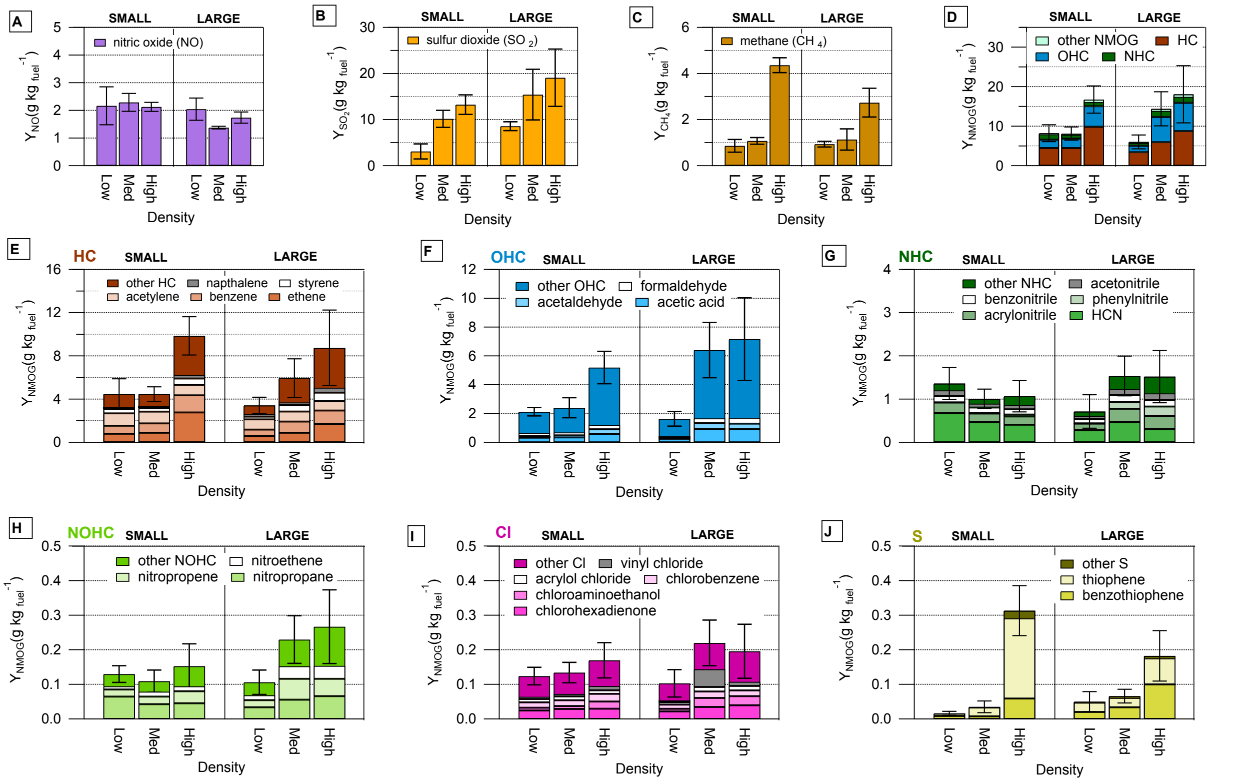

Figure S1. Gas yields (Y, g of gas per kg of dry fuel) of (A) NO, (B) SO_2, (C) CH_4 as measured by the FTIR, and (D) summed non-methane organic gases (NMOG) as measured by both the FTIR (for select gases) and PTR-MS. NMOGs are further categorized according to elemental composition: (E) HC, gases containing only carbon and hydrogen, (F) OHC, oxidized HC, (G) NHC, nitrogen-containing HC, (H) NOHC, nitrogen and oxygen HC, (I) Cl, chlorine-containing HC, and (J) S, sulfur-containing HC. Contributions from high-yield NMOGs, for example, are shown as lighter colored and patterned portions of total yield bars. Yields are shown as a function of surrogate size (small and large) and density. Relative standard deviations of 30 % and 40 % were applied to the yields for the large, medium density (LM) and large, high density (LH) surrogate yields (estimated from corresponding organic gas yields determined from triplicate FTIR measurements). All other error bars show the standard deviation of three replicate measurements.

Sensitivity of Positive Matrix Factorization (PMF) Solutions to Error Matrix.

When in the exploratory stage of performing PMF analyses we found that the PMF objective function ($Q/Q_{expected}$) was typically around 30 depending on what surrogate dataset was being analyzed. The objective function is a measure of how well the PMF solution fits the input dataset and is calculated as the ratio of the error (arising from the model fit with input data) to the known uncertainties with a value of 1 indicating a perfect fit and values above or below 1 indicating underestimates or overestimates of uncertainties, respectively.[1] We ultimately multiplied error matrices by a scalar of 9 to decrease the value of the objective function such that it was close to one. Yan, et al. (2016) explains that changes in the objective function, either as a function of number of factors or rotational transformations, can be useful in finding local minima in PMF solutions, but the absolute value of the objective function may not clearly indicate an optimal solution.[2] Below we explain how we prepared the error matrix and then present a case study demonstrating limited changes in PMF solutions as a function of error matrix preparation for a small high-density surrogate.

We prepared the error matrix in the following steps:

(1) Generate a matrix of ion count rate uncertainties (σ_{cps}) assuming a Poisson distribution (of the ion count rate, R_{cps}) following Equation S1.

$$\sigma_{cps} = \sqrt{R_{cps}} \qquad \text{(S1)}$$

(2) Divide these uncertainties by the sensitivity (cps per nmol mol^{-1}) for the corresponding NMOG ($\sigma_{nmol/mol}$). This step provides an uncertainty matrix in units of nmol mol^{-1}.

(3) Apply a minimum uncertainty value of 0.01 nmol mol^{-1} (close to the limit of detection for many NMOGs we have calibrated for directly) to any uncertainty values below that threshold.

(4) Multiply the error matrix by a factor of 9.

(5) Multiply the error for benzene by a factor of 10.

Figure S2A shows the relationship between the standard deviation of the ion signal versus the ion signal for the 201 NMOGs highlighted in this study for a 10 second measurement during the combustion of a small high density surrogate.

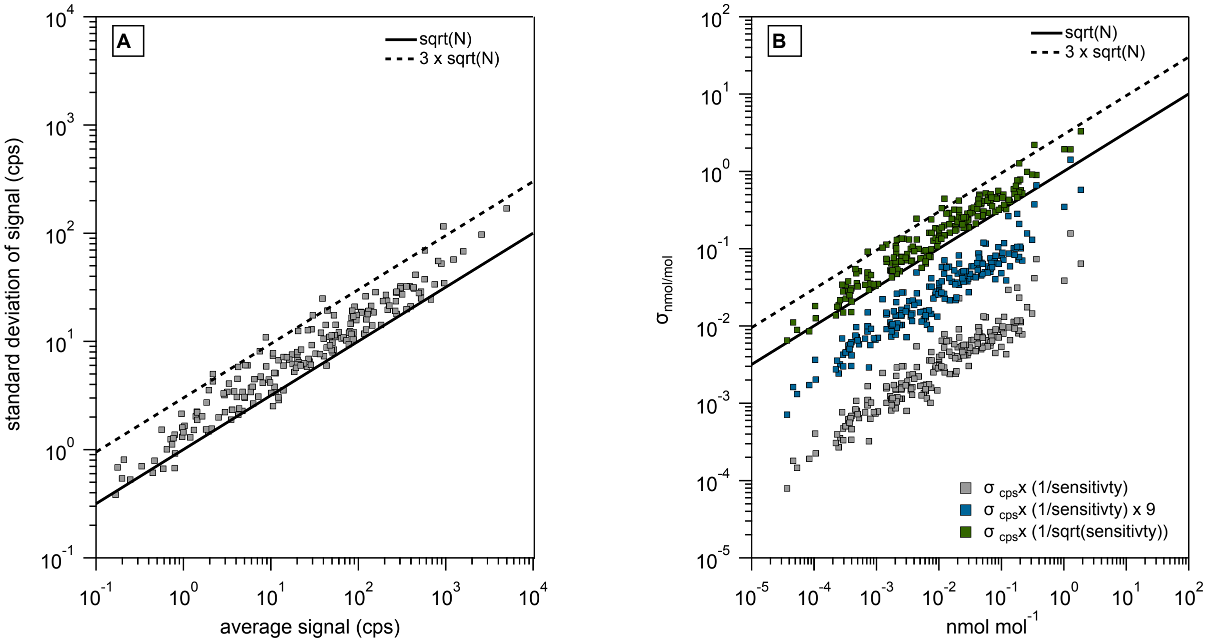

Figure S2. (A) Standard deviation of 201 NMOG ion signals measured from a 10 second period during a small high density surrogate combustion experiment plotted against the average of the signal. The solid

black line shows the square root of the average signal (functional form of uncertainties expected from counting statistics), and the dotted line shows three times the square root of the average to show the range that the measurements fall within. (B) The average ion signals in panel A are divided by ion-specific sensitivities to produce the mole fraction shown on the x-axis. The σ_{cps} is calculated following Equation S1 and the sensitivity is applied three different ways to create the three distributions of colored markers as described in the legend. Note that the blue markers do not include the factor of 10 applied to the error for benzene.

The standard deviations of the ion signals measured from our instrument follow the distribution expected for uncertainties from counting statistics ($\sqrt{N}$) within a factor of three. Yuan, et al. (2016) found standard deviations to fall within a factor of 2 of the expected $\sqrt{N}$ distribution and attributed higher than expected values to high-resolution peak fitting errors.[3] Other studies have multiplied counting statistics errors by a factor of 1.28 to account for analytical uncertainty and added additional error resulting instrument noise uncertainties.[2, 4]

In our study, we divided the uncertainties in ion counting rates by NMOG-specific sensitivities (cps per nmol mol^{-1}) to obtain a matrix of $\sigma_{nmol/mol}$ that we used as the error matrix for PMF. We ultimately decided on an error matrix for PMF represented by $\sigma_{nmol/mol}$ multiplied by 9 with a factor of 10 applied to the $\sigma_{nmol/mol}$ for benzene (Figure S2B, blue markers). Figure S2B shows three different ways we calculated $\sigma_{nmol/mol}$ for the 201 NMOGs as a function of mole fraction corresponding to the ion signals in Figure S2A. The blue markers representing the uncertainties we used in the error matrix fall in between the upper bound shown by the green markers and the lower bound shown by the gray markers. These bounds provide a range for us to test the sensitivity of our PMF solution to the treatment of the error matrix.

We evaluate the sensitivity of the PMF solutions to the treatment of the error matrix by (1) performing correlations of the factor mass spectral profiles and (2) performing correlations of the factor time series to understand how factor NMOG composition may have changed and how that may have affected PMF factor time series. We use a single experiment for a small high density surrogate as a case study. We test four treatments of the error matrix (each treatment in Table S1 and S2 corresponds to the number below):

(1) dividing the σ_{cps} by the sensitivity (gray markers in Figure S2B),

(2) dividing the σ_{cps} by the sensitivity and multiplying by 9 (blue markers in Figure S2B),

(3) dividing the σ_{cps} by the sensitivity and multiplying by 9 and applying a factor of 10 to the error for benzene (what we ultimately decided upon as the method for constructing the error matrix), and

(4) dividing the σ_{cps} by the square root of the sensitivity (green markers in Figure S2B).

The values for the objective function ($Q/Q_{expected}$) for treatments 1, 2, 3, and 4 were 33.0, 2.7, 2.6, and 0.12, respectively.

Table S1 shows the r^2 correlation coefficients for linear regressions of PMF mass spectral profiles. Tight correlations (> 0.95) for all PMF factors across the range of tested error matrices show that the PMF factor NMOG composition is insensitive to the treatment of the error matrix (within the ranges we tested). Table S2 shows the r^2 correlation coefficients for linear regressions of PMF factor time series. PMF factor time series were most affected by error treatments 3 and 4

when compared to error treatment 1. Time series correlations are still relatively high (around 0.9), and we expect the impact on our results related to PMF factor yields to be minimal.

Table S1. PMF Factor Mass Spectral Profile Correlations. The numbers (1, 2, 3, and 4) in the row and column labeled "error treatment" correspond to the four numbered error treatments listed above in the text.

	Mass Spectral Profile Correlations											
	Wood				Synthetic Polymer				Mixed Fuel Char			
Error Treatment	1	2	3	4	1	2	3	4	1	2	3	4
1		0.99	0.98	0.99		0.99	0.96	0.97		0.99	0.99	0.99
2			0.99	0.99			0.99	0.99			0.99	0.99
3				0.99				0.99				0.99
4												

Table S2. PMF Factor Time Series Correlations.

	PMF Factor Time Series Correlations		
	Wood	Synthetic Polymer	Mixed Fuel Char

Error Treatment	1	2	3	4	1	2	3	4	1	2	3	4
1		0.99	0.97	0.98		0.95	0.89	0.92		0.99	0.98	0.98
2			0.98	0.99			0.98	0.96			0.97	0.99
3				0.98				0.99				0.98
4												

For future studies we do not recommend multiplying the error matrix by a scaler to achieve a desired objective function value. The three-factor solution in our study was practically insensitive to the range of scaler values we applied to the error matrix once converting ion counts per second to nmol mol^{-1}. Multiplying the benzene error by 10 had small, but noticeable effects on the PMF factor profiles and time series for the three-factor solution in our study. Other studies have effectively downweighted high signal ions by removing the monoisotopic mass-to-charge from the PMF analyses altogether, but leaving their isotopic contributions.[5] For future work we will constrain PMF analyses of mixed fuel surrogate combustion with mass spectral profiles obtained from the combustion of individual components.

Additional Investigations of Seven-Factor PMF Solutions.

We investigated PMF solutions with up to seven factors to explain $NMOG_{PTR}$ production from the surrogates. Small changes in the PMF objective function were observed above three factor solutions. For some surrogates, factor time series correlations resulted in good agreement with other chemical species besides HCN and HCHO measured by the FTIR including ethene, NO, and ethane (see Figure S3). Despite good agreement of some factors with different FTIR

chemical species with increasing factor solutions, the mass spectra produced from these factors did not correlate well with factors that correlated with the same FTIR species for different surrogates. For instance, a factor's mass spectral profile produced from the SL surrogate would typically not correlate well with the factor's profile from the SH surrogate despite both factor time series correlating with NO. Because increasing the number of factors above three introduced complexities in data interpretation without adding well-defined value to the analysis, we constrained our PMF factor analysis to three factors.

In Figure S3 we show an example of an analysis we performed for a seven-factor solution from an SL surrogate. Seven-factor solutions generally produced factors that we interpreted as "splitting" from the three main factors (wood, synthetic polymer, and mixed fuel char) we based our final analyses on. For instance, Wood 1, 2, and 3 in Figure S3 appear to be split from the Wood factor from our three-factor solution. Similarly, Mixed Fuel Char (MFC) 1, 2, and 3 in Figure S3 appear to be split from the Mixed Fuel Char factor from our three-factor solution.

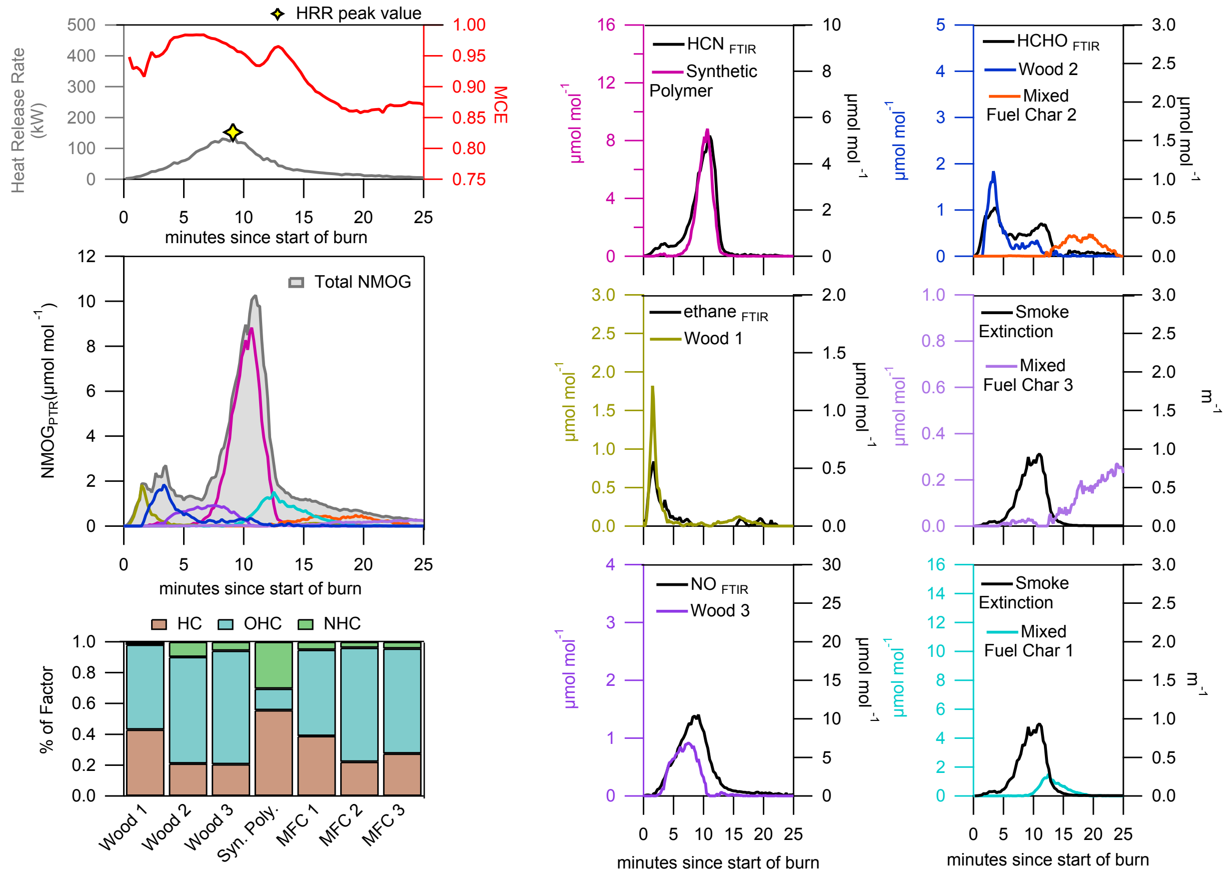

Small Low Density Surrogate (SL)

Figure S3. Seven factor solution for an SL surrogate PMF analysis.

The source of the variability in correlations of the three-factor PMF factor mass spectral profiles (wood, synthetic polymer, and mixed fuel char) across surrogates in our final analyses likely arises from small differences in combustion of the surrogate component materials that results in different NMOG emissions. For instance, in Figure S3 the synthetic polymer factor appears as a single factor for the SL surrogate whereas it is split into three different factors for the SH surrogate (Figure S4). In Figure S5 we show how images of the surrogate combustion correspond to PMF factor appearance for the SL surrogate seven factor solution.

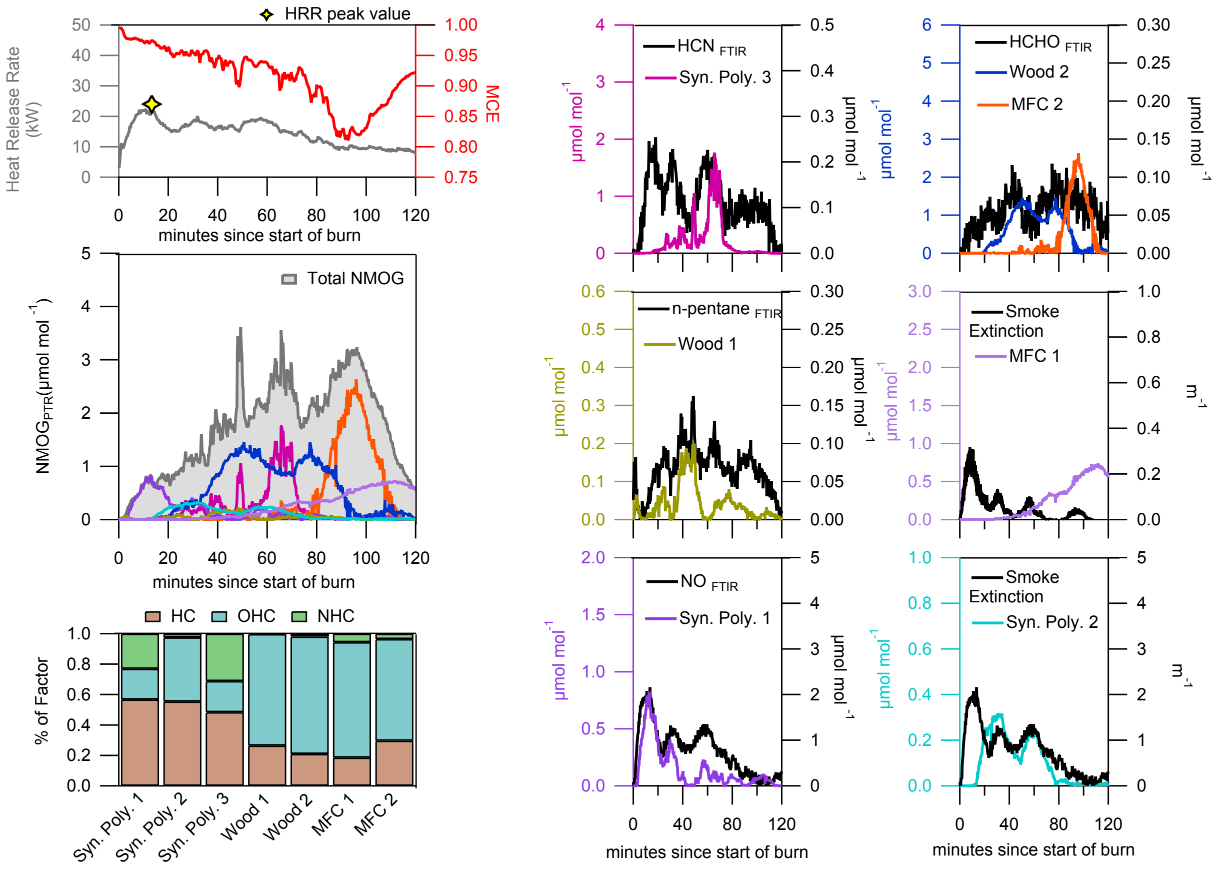

Small High Density Surrogate (SH)

Figure S4. Seven factor time series solution for an SH surrogate PMF analysis.

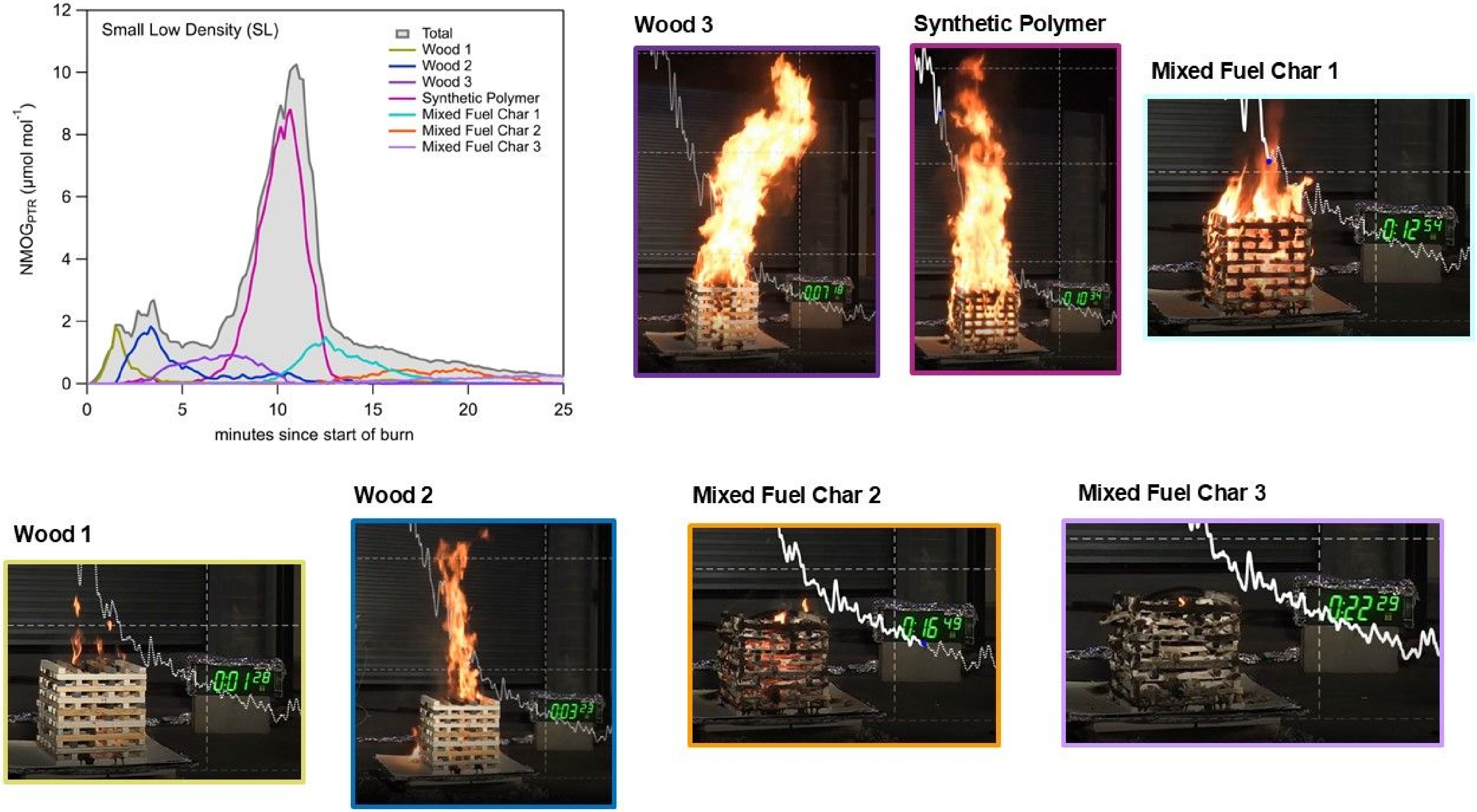

Figure S5. Pictures of the surrogate corresponding to when factors peak for the seven-factor solution for the SL surrogate.

Correlations for Three-Factor PMF Solution.

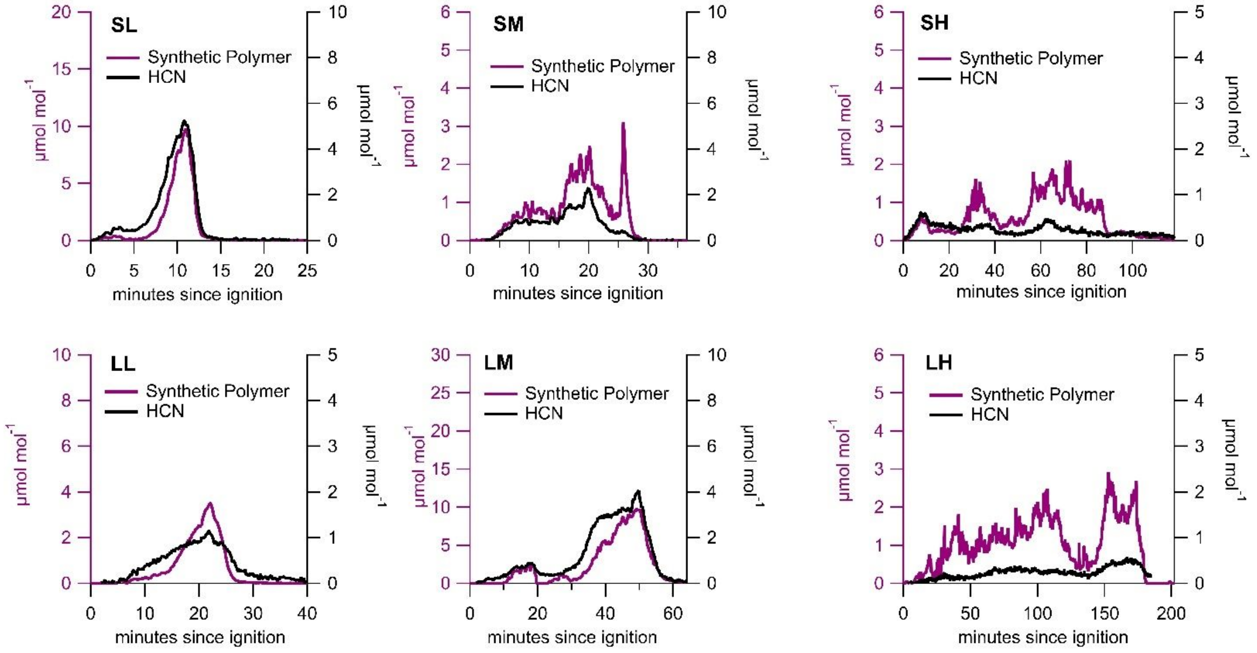

Figure S6. Time series correlations of the synthetic polymer factor with the HCN tracer for six different surrogates.

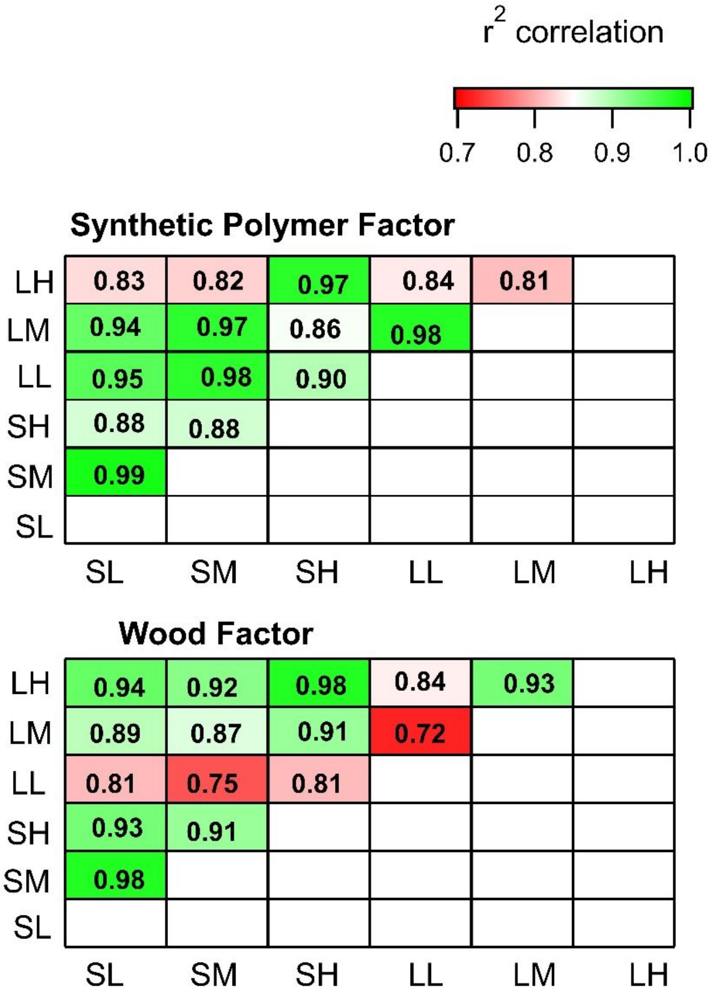

Figure S7. Correlation matrices of the synthetic polymer and wood factor mass spectral profiles, from the final 3-factor PMF solutions, across the various surrogate configurations. Correlations of the mixed fuel char factor are not shown since they were all generally at or below an $r^2 < 0.6$.

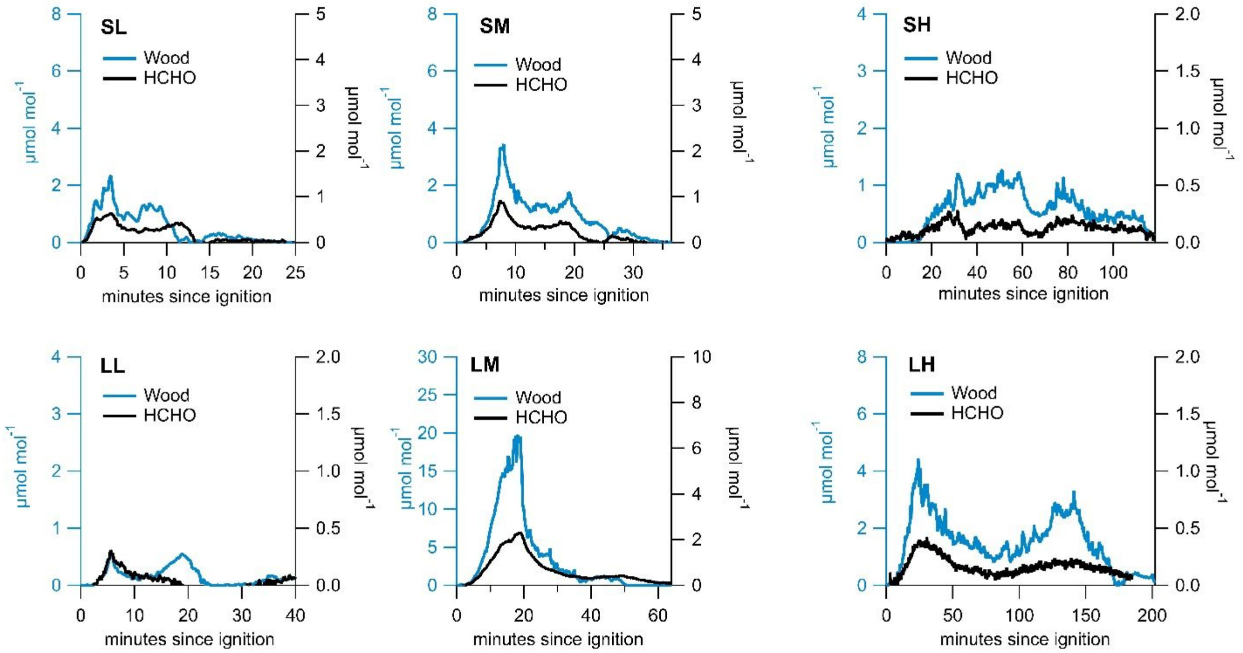

Figure S8. Time series correlations of the wood factor with the HCHO tracer for six different surrogates.

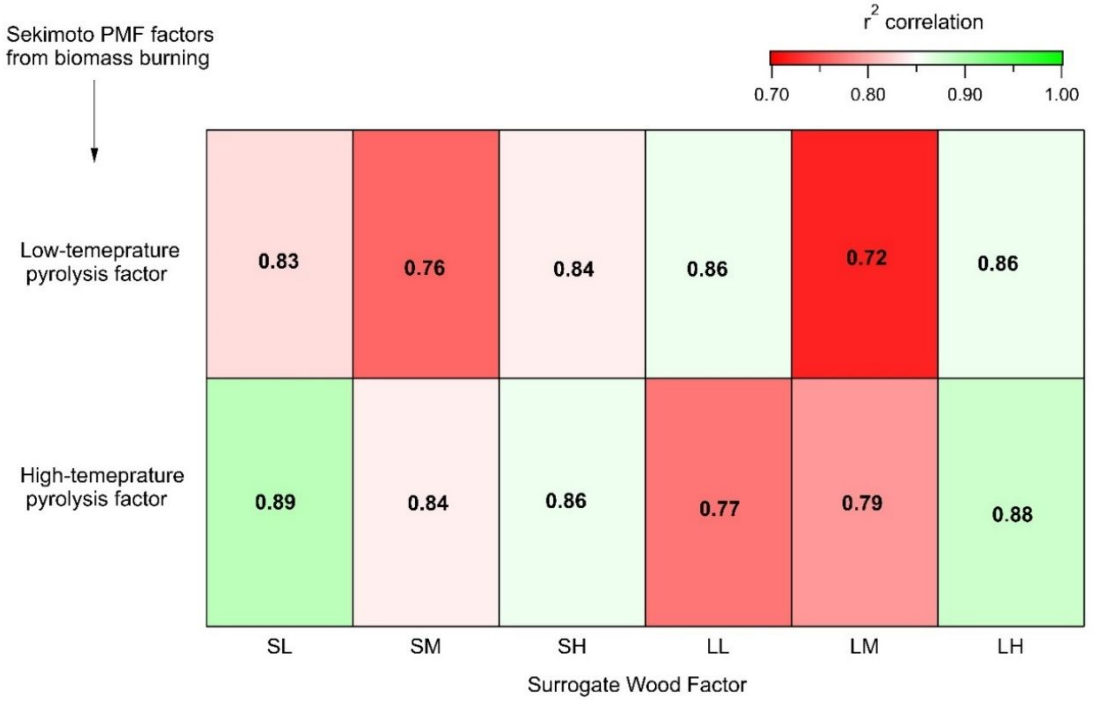

Figure S9. Correlations of wood mixed fuel char factors with "high" and "low" temperature pyrolysis factors from Sekimoto et al (2018). Numbers inside the boxes show the Pearsons r^2 coefficient value for correlation of the Sekimoto mass spectral factor profile with the wood factor from the corresponding surrogate. We only correlated species that appeared in mass spectral profiles for both the Sekimoto factors and the factors measured in this study. Additionally, we removed benzene from the factors because factor correlations improved considerably.

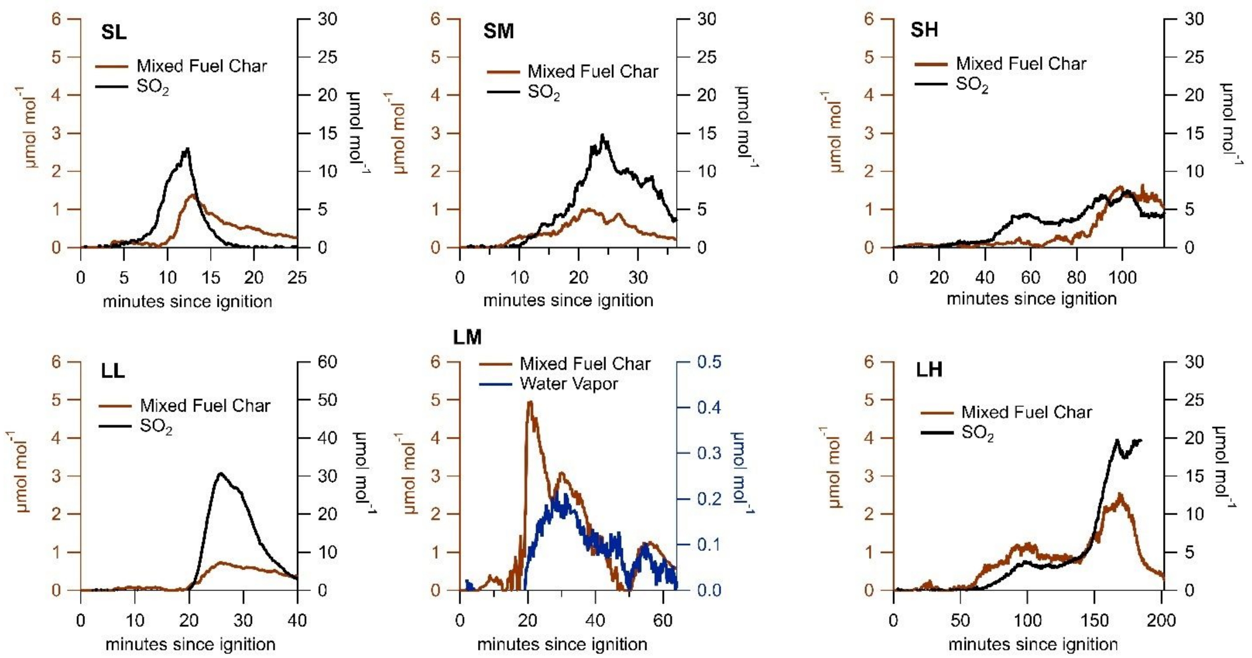

Figure S10. Time series correlations of mixed fuel char factor with the SO_2 tracer for five different surrogates and water vapor for one of the surrogates.

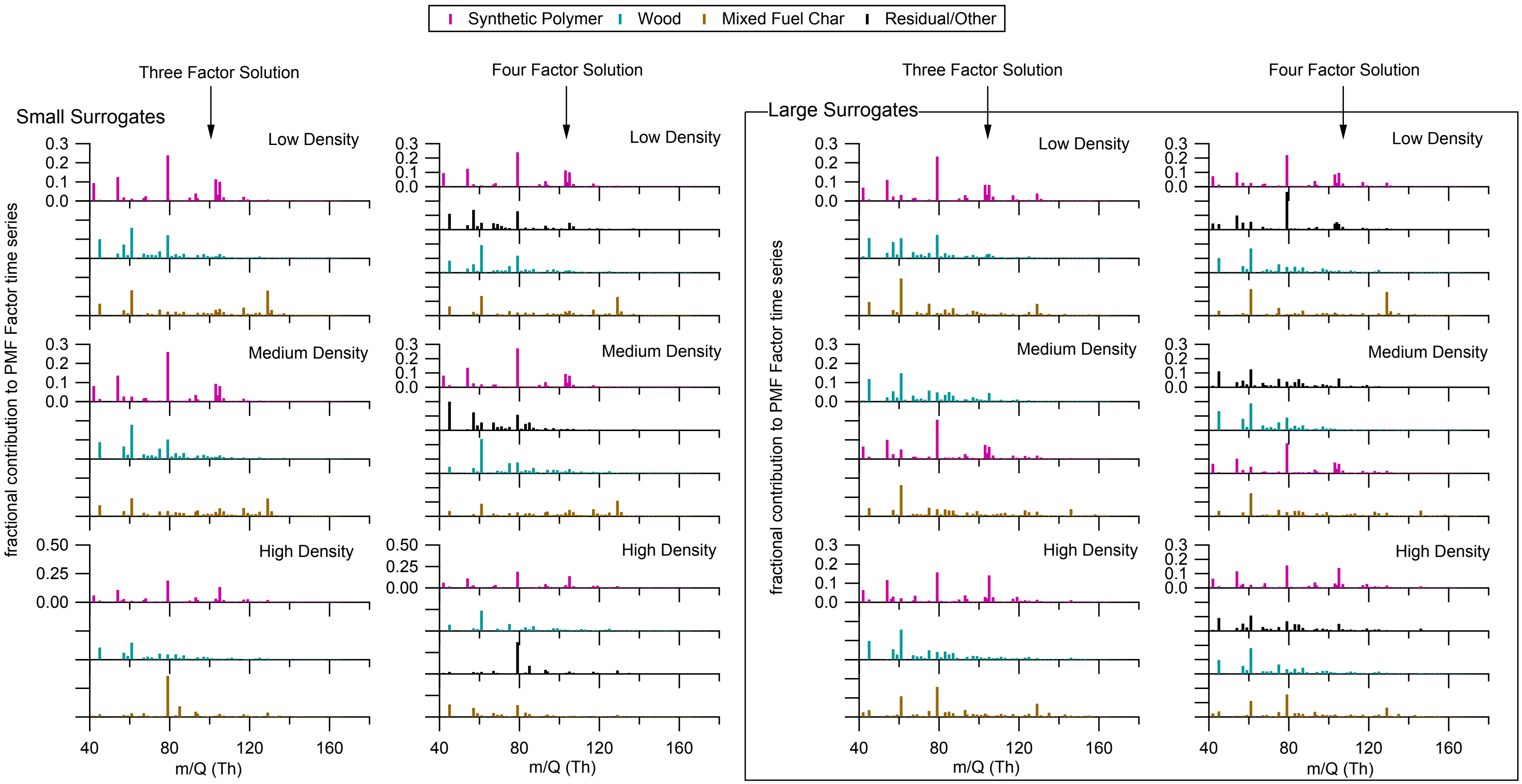

Figure S11. Factor splitting of three factor solutions to four factor solutions.

Additional PMF Factor Time Series Analyses.

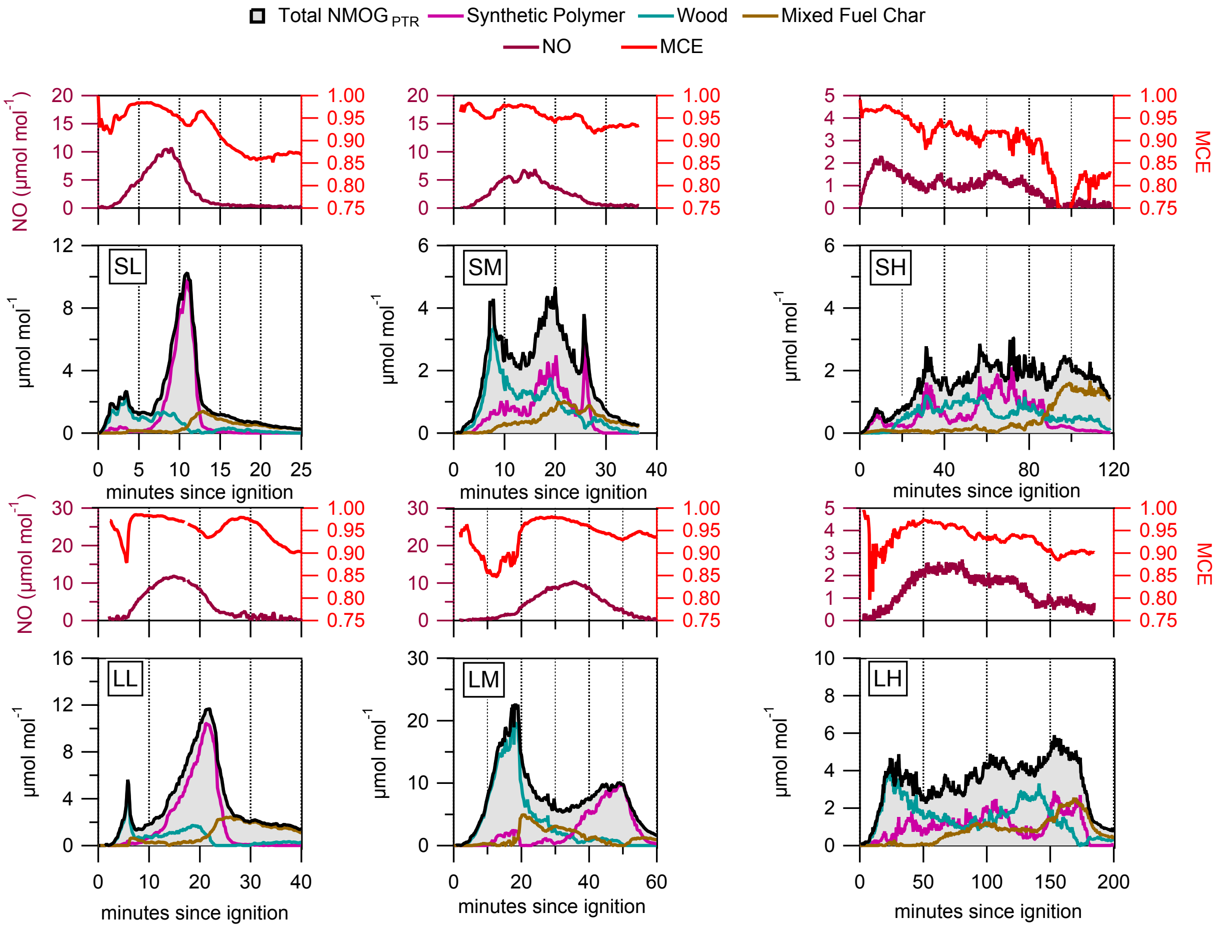

Figure S12. Time series of $NMOG_{PTR}$ factors plotted with nitric oxide (NO) concentrations (left axis of upper panel) and modified combustion efficiency (MCE). Surrogate identity (S = small, L= large size and L = low, M = medium, and H = high density) is shown in the NMOG time series panel and the corresponding time series for NO and MCE is plotted directly above. NO concentrations are typically elevated just prior to high concentrations of the synthetic polymer factor indicating lower temperature pyrolysis is generating NMOGs from polymer combustion. We note that although the CO_2 concentration is not shown in the figure it follows the NO concentration.

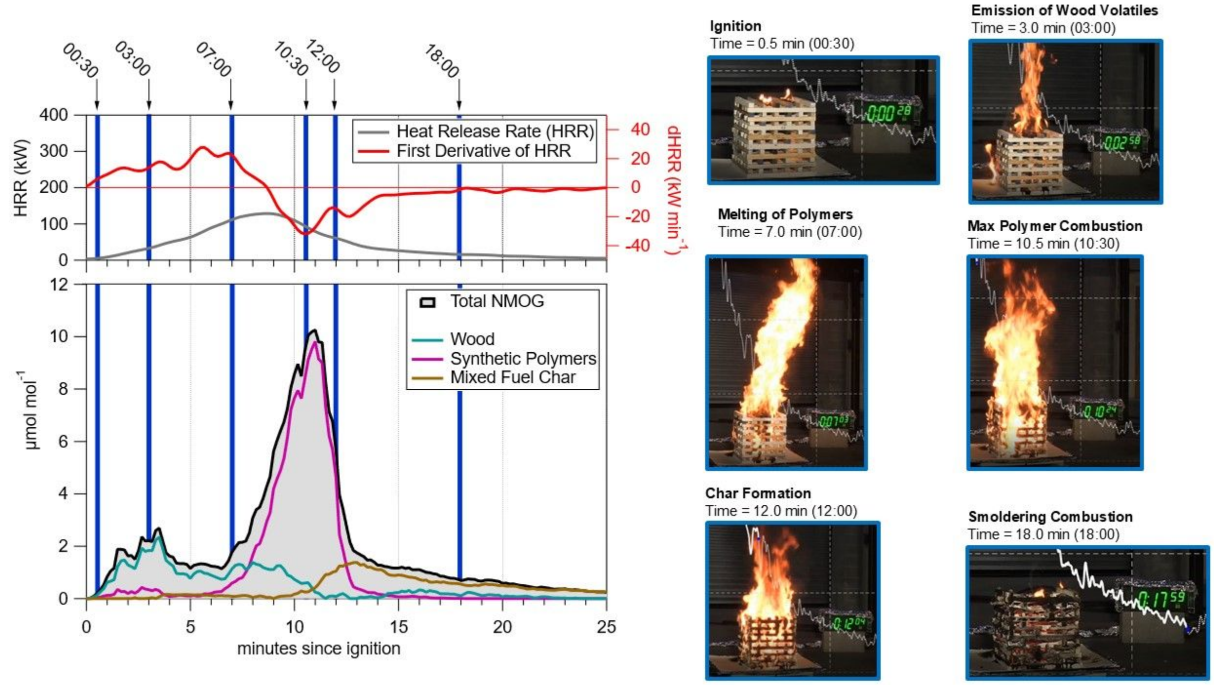

Figure S13. Pictures corresponding to the PMF time series of an SL surrogate to show visual indicators of combustion. In particular, pools of melted ABS can be seen at the base of the surrogate in the frame corresponding to "max polymer combustion".

PMF Factor Yields.

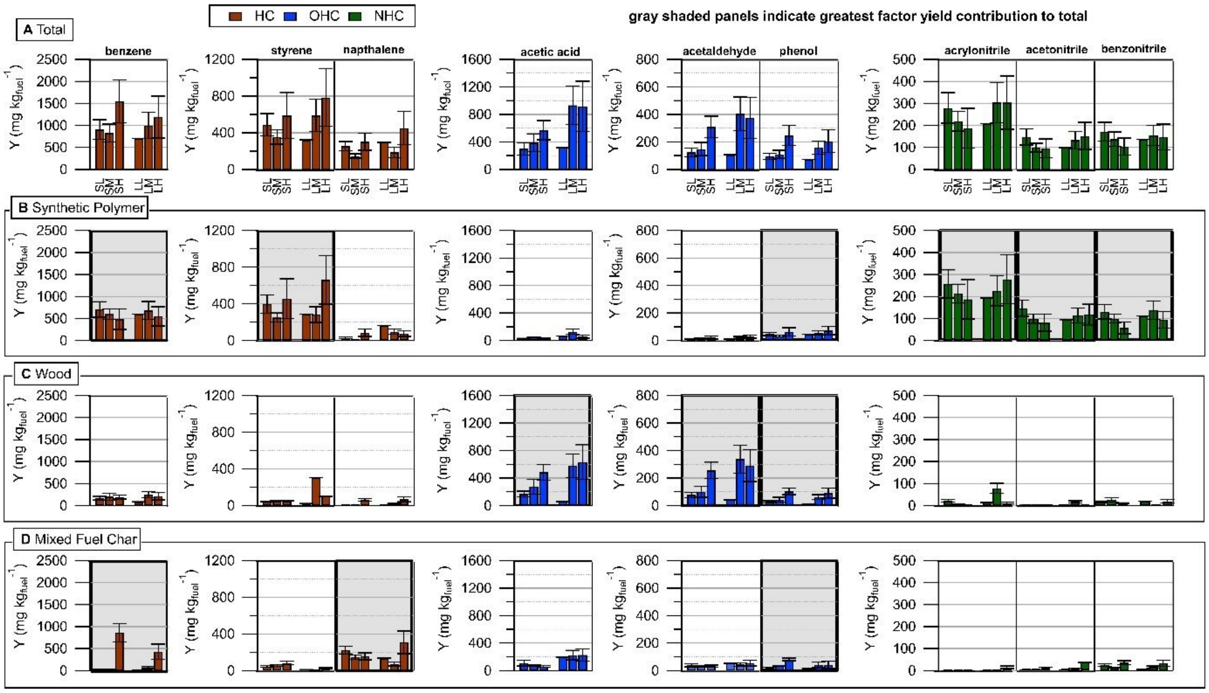

Figure S14. Total NMOG yields for select species representing three different chemical classes (HC, OHC, and NHC) as a function of surrogate density and size. The first three columns correspond to small surrogates and the last three to large surrogates each in order of least to most densely packed. The axes directly below an NMOG in the first row correspond to the yield associated with the factor corresponding to the boxed area. For instance, the yields in the second row (B) correspond to the synthetic polymer factor, the third row (C) to the fresh wood factor, and the fourth row (D) to the charred wood factor. Gray shaded and bolded panels within a given column show that the factor in the corresponding row was most responsible for the total yield. Phenol had nearly equal contributions from all factors to the total yield.

References

(1) Ulbrich, I. M.; Canagaratna, M. R.; Zhang, Q.; Worsnop, D. R.; Jimenez, J. L. Interpretation of organic components from Positive Matrix Factorization of aerosol mass spectrometric data. *Atmospheric Chemistry and Physics* **2009**, *9* (9), 2891-2918. DOI: 10.5194/acp-9-2891-2009.

(2) Yan, C.; Nie, W.; Äijälä, M.; Rissanen, M. P.; Canagaratna, M. R.; Massoli, P.; Junninen, H.; Jokinen, T.; Sarnela, N.; Häme, S. A. K.; et al. Source characterization of highly oxidized multifunctional compounds in a boreal forest environment using positive matrix factorization. *Atmospheric Chemistry and Physics* **2016**, *16* (19), 12715-12731. DOI: 10.5194/acp-16-12715-2016.

(3) Yuan, B.; Koss, A.; Warneke, C.; Gilman, J. B.; Lerner, B. M.; Stark, H.; De Gouw, J. A. A high-resolution time-of-flight chemical ionization mass spectrometer utilizing hydronium ions (H_3O^+ ToF-CIMS) for measurements of volatile organic compounds in the atmosphere. *Atmospheric Measurement Techniques* **2016**, *9* (6), 2735-2752. DOI: 10.5194/amt-9-2735-2016.

(4) Zhang, Y.; Peräkylä, O.; Yan, C.; Heikkinen, L.; Äijälä, M.; Daellenbach, K. R.; Zha, Q.; Riva, M.; Garmash, O.; Junninen, H.; et al. A novel approach for simple statistical analysis of high-resolution mass spectra. *Atmospheric Measurement Techniques* **2019**, *12* (7), 3761-3776. DOI: 10.5194/amt-12-3761-2019.

(5) Li, H.; Canagaratna, M. R.; Riva, M.; Rantala, P.; Zhang, Y.; Thomas, S.; Heikkinen, L.; Flaud, P.-M.; Villenave, E.; Perraudin, E.; et al. Atmospheric organic vapors in two European pine forests measured by a Vocus PTR-TOF: insights into monoterpene and sesquiterpene oxidation processes. *Atmospheric Chemistry and Physics* **2021**, *21* (5), 4123-4147. DOI: 10.5194/acp-21-4123-2021.
